# Salvianolic Acid B: A Review of Pharmacological Effects, Safety, Combination Therapy, New Dosage Forms, and Novel Drug Delivery Routes

**DOI:** 10.3390/pharmaceutics15092235

**Published:** 2023-08-29

**Authors:** Guannan He, Guangfeng Chen, Weidong Liu, Dongxue Ye, Xuehuan Liu, Xiaodong Liang, Jing Song

**Affiliations:** 1 Shandong University of Traditional Chinese Medicine, Jinan 250355, China; 2021110111@sdutcm.edu.cn (G.H.); ray_liuwd@163.com (W.L.); yedongxuesss@163.com (D.Y.); 2 Department of Geriatrics, Affiliated Hospital of Shandong University of Traditional Chinese Medicine, Jinan 250355, China; shang3han@126.com; 3 Experimental Center, Shandong University of Traditional Chinese Medicine, Jinan 250355, China; lxh1055942457@163.com; 4 Shandong Yuze Pharmaceutical Industry Technology Research Institute Co., Ltd., Dezhou 251200, China

**Keywords:** salvianolic acid B, pharmacological effects, safety, combination therapy, new dosage forms, novel drug delivery routes

## Abstract

Salvianolic acid B is extracted from the roots and rhizomes of Danshen (*Salvia miltiorrhiza* Bge., family Labiatae). It is a water-soluble, weakly acidic drug that has demonstrated antitumor and anti-inflammatory effects on various organs and tissues such as the lung, heart, kidney, intestine, bone, liver, and skin and protective effects in diseases such as depression and spinal cord injury. The mechanisms underlying the protective effects of salvianolic acid B are mainly related to its anti-inflammatory, antioxidant, anti- or pro-apoptotic, anti- or pro-autophagy, anti-fibrotic, and metabolism-regulating functions. Salvianolic acid B can regulate various signaling pathways, cells, and molecules to achieve maximum therapeutic effects. This review summarizes the safety profile, combination therapy potential, and new dosage forms and delivery routes of salvianolic acid B. Although significant research progress has been made, more in-depth pharmacological studies are warranted to identify the mechanism of action, related signaling pathways, more suitable combination drugs, more effective dosage forms, and novel routes of administration of salvianolic acid B.

## 1. Introduction

**Figure 1:**
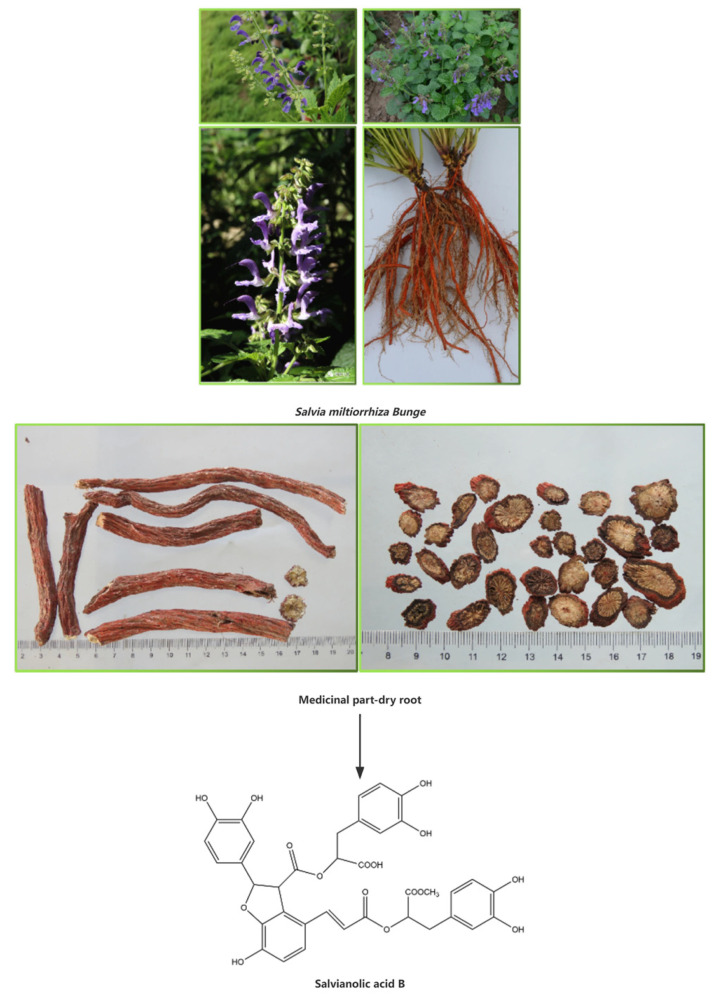
The original plants and herbs of Danshen and the chemical structure of salvianolic acid B.

Danshen, also known as Salviae Miltiorrhizae Radix et Rhizoma, is widely used in traditional Chinese medicine for the treatment of conditions such as heartache, stomachache, joint pain, and irregular menstruation. The pharmacodynamic material basis of Danshen is mainly represented by water-soluble components such as salvianolic acid [1,2], with salvianolic acid B being the most abundant water-soluble component [3]. The molecular formula of salvianolic acid B is C36H30O16, and its relative molecular weight is 718.62. It is formed through the condensation of three molecules of Danshensu and one molecule of caffeic acid, and its stability in water is poor (Figure 1). High-speed counter-current chromatography can be performed yielding 342 mg salvianolic acid B at 98% purity from 500 mg of the crude extract separation [4]. Another study found that high-performance counter-current chromatography 1.5 g of crude sample was processed and 475 mg 96.1% pure salvianolic acid B was harvested with a 42.8% recovery [5]. The content of salvianolic acid B can reach 85.50% by taking alcohol aqueous extraction and adding flocculant chitosan, and then purified by macroporous adsorbent resin [6]. Salvianolic acid B was separated and purified by microbial transformation together with the chromatography of microsphere resin, and the purity of salvianolic acid B reached up to 95% at a yield of 62% [7]. The salvianolic acid B content after 30% ethanol reflux extraction was 57.7% [8]. Using the ultrasound-assisted method extraction of salvianolic acid B, the yield was 5.17 mg/g (33.93 mg/g) higher than that from the conventional refluxing method (28.76 mg/g), and a higher extraction yield can be achieved over a shorter time period and at a lower temperature [9]. Salvianolic acid B is also minimally lost during ultrafiltration [10].

**Figure 2:**
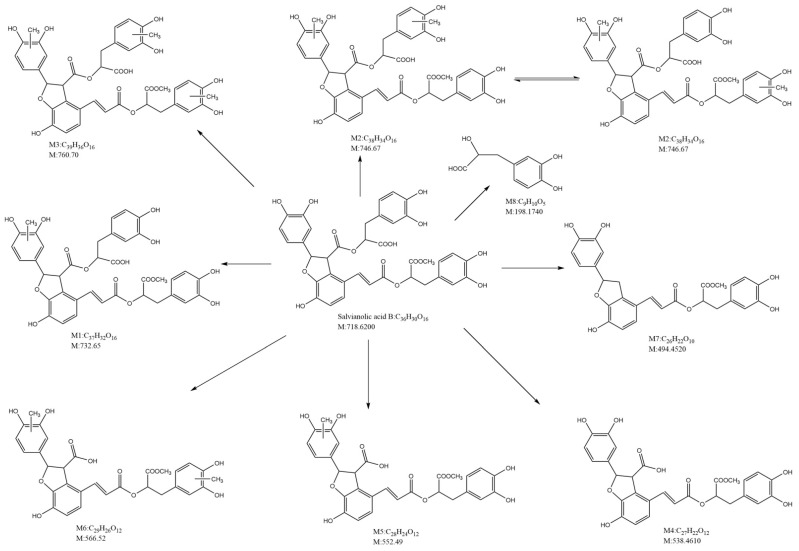
Possible metabolites of salvianolic acid B. The figure is adapted from [12].

To transport salvianolic acid B or facilitate its diffusion in rats, the ileum was shown to be the best absorption section [11]. In a study, high-performance liquid chromatography–tandem quadrupole time-of-flight mass spectrometry (HPLC-Q-TOF-MS/MS) revealed that salvianolic acid B was mainly metabolized through methylation and ester bond hydrolysis and majorly eliminated through biliary excretion in rats [12] (Figure 2). In another study, ultra-high-performance liquid chromatography with linear ion trap–orbitrap mass spectrometry (UHPLC-LTQ-Orbitrap MS) revealed 15 metabolites of salvianolic acid B in rats, with the main biotransformation pathways being five-membered ring cracking, ester bond cleavage, decarboxylation, dehydroxylation, hydrogenation, methylation, sulfonation, glucuronidation, and their compound reactions [13]. LC–MS/MS showed that after salvianolic acid B was given in conscious and freely moving rats, the AUCs were 5030 ± 565 and 582 ± 222 min μg/mL for intravenous (100 mg/kg) and oral (500 mg/kg) doses, respectively, and the oral bioavailability of salvianolic acid B in rats was 2.3% [14]. The gradient elution HPLC method showed that after salvianolic acids (180 mg/kg, oral; 9 mg/kg, intravenous) were given, the AUCs of salvianolic acids B were 1680 ± 670 and 7840 ± 1140 ng/mL·h, respectively. The bioavailability of salvianolic acids B in dogs was only 1.07 ± 0.43% [15]. An in-depth understanding of the absorption, metabolic pathways and profiles, and bioavailability of salvianolic acid B may facilitate further basic research on its pharmacological effects and mechanisms. Salvianolic acid B is a strong active ingredient used in herbal medicine and has many pharmacological activities. It has been used in the treatment of liver diseases [16,17,18], kidney diseases [19], brain and neurological diseases [20], heart diseases [21,22], skin diseases [23], vascular diseases [24], bone diseases [25,26], lung diseases [27], tumors [28], diabetes [29,30], and diabetes-related complications [31,32].

As a natural ingredient, salvianolic acid B is safe and less toxic and has strong activity; however, its poor stability limits its use to some extent [33]. Improving the stability of salvianolic acid B and increasing its production efficiency are necessary approaches to expanding its clinical applications. Identifying new dosage forms and routes of administration of salvianolic acid B and combining it with other drugs can increase therapeutic efficacy. In particular, new dosage forms and routes of administration can help achieve targeted delivery and have a sustained release effect. Therefore, these three approaches hold great promise for the further research and development of salvianolic acid B. This review systematically summarizes the pharmacological effects, safety profile, combination therapy potential, and new dosage forms and delivery routes of salvianolic acid B to provide comprehensive information for further studies.

## 2. Pharmacological Effects

### 2.1. Effects on the Tumor

Salvianolic acid B can directly bind to ubiquitin carboxyl-terminal hydrolase 2 (USP2) and inhibit its deubiquitinating activity in colon cancer cells RKO, thus promoting the ubiquitin–proteasome pathway degradation of programmed cell death ligand 1 (PD-L1) proteins and enhancing the killing activity of T cells against tumor cells, eventually exerting antitumor effects. The tumor suppression rate of salvianolic acid B at concentrations of 10 and 20 mg/kg has been reported to be 43.4% and 63.2%, respectively, in mice inoculated with colon cancer cells MC38 [34]. In addition, salvianolic acid B can activate the expression of autophagy-associated proteins light chain 3-II type (LC3-II) and autophagy-associated protein 5 homolog (Atg5) in HCT116 colon cancer cells by inhibiting the protein kinase B (Akt)/mammalian rapamycin target protein (mTOR) signaling pathway. Therefore, salvianolic acid B can induce autophagy, thereby inhibiting the proliferation of colon cancer cells [35].

Epithelial–mesenchymal transition (EMT) is an important transformational process in cancer, which can enhance the flexibility and invasive ability of tumor cells [36]. Salvianolic acid B can inhibit the TGF-β1-induced growth of A549 human non-small-cell lung cancer (NSCLC) cells by inactivating the phosphorylation of mitogen-activated protein kinase (MAPK) and Smad2/3. In addition, it can inhibit EMT and cell migration, hamper cell cycle progression, and induce autophagy and apoptosis in A549 cells [37]. In the NSCLC cell lines NCI-H2030 and NCI-H1650, salvianolic acid B can inhibit pyruvate kinase M2 (PKM2)-mediated metabolic reprogramming, downregulate the expression of the metabolic reprogramming-related genes lactate dehydrogenase A (LDHA) and glucose transporter 1 (GLUT1), and inactivate EMT (markedly reduced transcription factors β-catenin expression as well as elevated transmembrane glycoproteins E-cadherin expression), thereby attenuating the metastasis of NSCLC [38].

In cisplatin-resistant AGS gastric cancer cells, salvianolic acid B can inhibit cell proliferation, induce apoptosis and oxidative stress, and reduce cisplatin resistance via the Akt/mTOR pathway by inhibiting EMT [39]. In addition, salvianolic acid B can specifically bind to mortalin, which is highly expressed in many cancers, and increase the degradation of mortalin proteasomes through ubiquitination, which upregulates reversion-inducing cysteine-rich protein with Kazal motifs (RECK) and inhibits signal transducer and activator of transcription 3 (STAT3), thereby reversing EMT and attenuating the migratory and invasive potential of HepG2 and HCCLM3 hepatocellular carcinoma cells [40]. In a study on rat models of 3-aminopropionitrile-induced thoracic aortic aneurysm, salvianolic acid B was found to inhibit the development of aneurysms and improve immune function by inhibiting the Janus kinase 2 (JAK2)/STAT3 signaling pathway, thus reducing malondialdehyde (MDA) and reactive oxygen species (ROS) levels, increasing superoxide dismutase (SOD) levels, and improving oxidative stress in rats. In addition, the expression of interleukin-1β (IL-1β), IL-6, IL-8, tumor necrosis factor-alpha (TNF-α), matrix metalloproteinase (MMP)-2, and MMP-9 was reduced, suggesting that salvianolic acid B inhibited the inflammatory response and reduced the production of MMPs [41].

In MDA-MB-231 and MDA-MB-468 triple-negative breast cancer cells, salvianolic acid B can upregulate the expression of the mesenchymal cell marker proteins N-cadherin and vimentin and the nuclear transcription factors p-Snail/Snail and p-Slug/Slug and downregulate the expression of the epithelial cell marker proteins E-cadherin and cytokeratin 19, eventually inhibiting EMT and attenuating the migratory, invasive, and adhesive abilities of tumor cells [42]. Ianni et al. demonstrated that salvianolic acid B downregulated MMP-9 in MDA-MB-231 cells and inhibited the invasive potential of the cell [43]. In a study, mice were inoculated with both 4T1 murine-derived breast cancer cells and MDA-MB-231 human-derived breast cancer cells. In these mice, salvianolic acid B blocked platelet-derived growth factor-B signaling by inhibiting the phosphorylation of platelet-derived growth factor receptor-β, inhibited the action of tumor cells on perivascular cells, promoted the structural integrity of tumor vessels, increased perivascular cell coverage (elevated αSMA+ and NG2+), promoted basement membrane integrity (elevated collagen IV+), enhanced tight junctions between endothelial cells, reduced vascular leakage, and promoted normalization of tumor vessels, eventually inhibiting tumor metastasis [44]. Salvianolic acid B exerts antitumor effects on MCF-7 human breast adenocarcinoma cells by enhancing apoptosis (increased expression of the apoptotic markers caspase-3 and P53), reducing oxidative stress (decreased MDA and increased glutathione (GSH) levels), reducing inflammation (decreased expression of TNF-α and MMP-8), and inhibiting angiogenesis (decreased expression of vascular endothelial growth factor (VEGF), cyclooxygenase-2 (COX-2), and cell cyclin D1). In mice injected with Ehrlich solid carcinoma cells, salvianolic acid B has been demonstrated to reduce tumor volume and increase median survival [45].

Salvianolic acid B inhibits the proliferative activity of MFE-280 endometrial cancer cells by inhibiting the phosphoinositide 3 kinase (PI3K)/Akt signaling pathway. In addition, it promotes the expression of the autophagy-related proteins Beclin1, LC3-II/LC3-I, and Atg7; decreases cellular mitochondrial membrane potential; and induces autophagy, apoptosis, and senescence of MFE-280 cells, eventually exerting antitumor effects [46].

In ID8 ovarian cancer cells, salvianolic acid B inhibited cell proliferation not only by downregulating the PI3K/Akt signaling pathways of six proteins, namely PI3K110β, PDPK1, p-PDPK1 (Ser241), pan-Akt, p-Akt1 (Ser473), and p-GSK3β (Y216), but also by downregulating the protein expression of phosphorylation (p)-IκBα/IκBα and p-p65/p65 of the nuclear factor kappa-B (NF-κB) signaling pathway. In vivo, salvianolic acid B can inhibit tumor growth in mice inoculated with ID8 cells [47,48].

**Figure 3:**
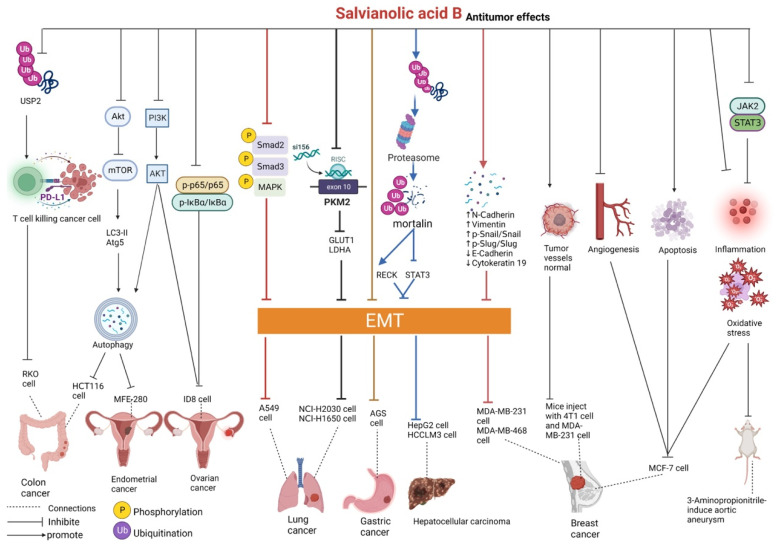
Antitumor mechanism of salvianolic acid B.

The findings of the abovementioned studies may introduce novel directions for further research (Figure 3 and Table 1). Altogether, salvianolic acid B exerts good protective effects against solid tumors, colon cancer, lung cancer, gastric cancer, liver cancer, thoracic aortic aneurysm, breast cancer, endometrial cancer, and ovarian cancer. Salvianolic acid B exerts antitumor effects primarily by inducing apoptosis, autophagy, and oxidative stress; enhancing the killing activity of T cells; inhibiting EMT and tumor cell proliferation; suppressing metabolic reprogramming; promoting anti-inflammation and the normalization of tumor vessels; and reducing the production of MMPs.

### 2.2. Effects on the Lung

Salvianolic acid B exerts remarkable therapeutic effects on pulmonary fibrosis [49]. In A549 human lung epithelial cells with TGF-β1-induced EMT, salvianolic acid B can downregulate fiber protein (FN) and collagen type I and suppress cell proliferation, thus exerting effective therapeutic effects against pulmonary fibrosis [50]. In addition, salvianolic acid B can prevent pulmonary fibrosis through anti-inflammatory and anti-fibrotic effects. A study demonstrated that salvianolic acid B inhibited not only lipopolysaccharide (LPS)-induced inflammation in THP-1 human acute monocytic leukemia cells (downregulated mRNA and protein expression of IL-1β and TNF-α) but also TGF-β1-induced proliferation of lung fibrosis model MRC-5 cells (decreased expression of alpha-smooth muscle actin (α-SMA) and collagen 1α1) [51]. In a study on rat models of bleomycin-induced idiopathic pulmonary fibrosis, nebulized inhalation of salvianolic acid B reduced the infiltration of lymphocytes and neutrophils, protected the basic structure of the lung from destruction, and inhibited the proliferation of fibrous tissue. These changes validate the antifibrotic effects of salvianolic acid B and can be attributed to its inhibitory effects on the inhibition of coagulation factor activation and the downregulation of protease-activated receptor-1 (PAR-1) and phospho-protein kinase C (p-PKC) [52].

In a study, staphylococcal pneumonia was induced in young rats via nasal dripping of *Staphylococcus aureus*. Salvianolic acid B reduced the levels of IL-1β, IL-6, TNF-α, MDA, and NF-κBp65; increased SOD levels; and improved inflammation in lung tissues in these rats [53].

**Figure 4:**
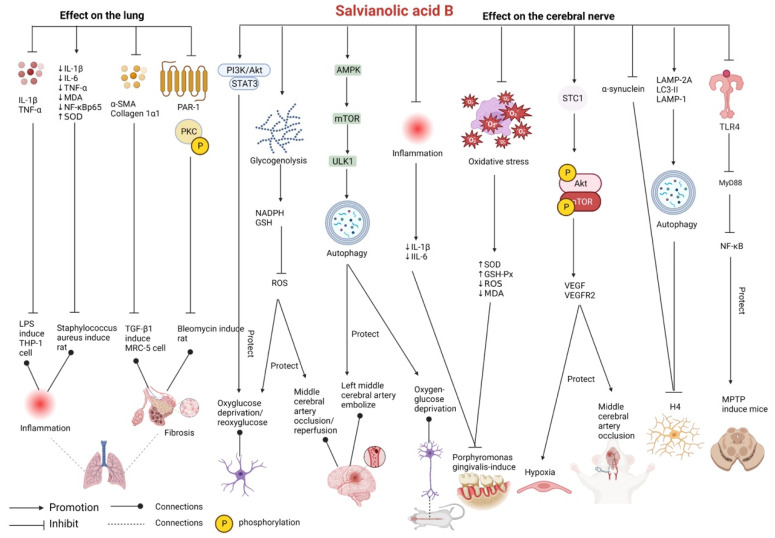
Mechanism of action of salvianolic acid B on lung and cerebral nerves.

The protective effects of salvianolic acid B against lung diseases, such as pulmonary fibrosis and pneumonia, have been investigated in both in vitro and in vivo studies. Salvianolic acid B inhibits the production of inflammatory cytokines including IL-1β and TNF-α and decreases the levels of fibrosis markers such as α-SMA and collagen. It exerts antifibrotic effects by inhibiting the activation of coagulation factors (Figure 4 and Table 2).

### 2.3. Effects on the Cerebral Nerve

Salvianolic acid B protects astrocytes by regulating the PI3K/Akt and STAT3 signaling pathways, inhibiting the release of ROS from astrocytes after oxygen–glucose deprivation/reperfusion injury, alleviating oxidative stress, increasing mitochondrial membrane potential and the release of BDNF and IGF1α (neurotrophic factors) from astrocytes, and decreasing intracellular calcium overload [54]. In mice with *Porphyromonas gingivalis*-induced periodontitis with cognitive impairment, salvianolic acid B can improve cognitive function by inhibiting neuroinflammation (decrease expression IL-1β and IL-6) and decreasing amyloid-β (Aβ) levels. In addition, it can alleviate oxidative stress, increase the activities of SOD and GSH-Px, decrease the levels of ROS and MDA, and upregulate the mRNA expression of the neurotrophic factors NGF and BDNF to protect against *Porphyromonas gingivalis*-induced memory impairment [55].

In an experiment on middle cerebral artery occlusion-induced ischemic stroke rats, salvianolic acid B attenuated ischemic brain injury and neurological injury by increasing stanniocalcin 1 (STC1), thus inducing Akt/mTOR phosphorylation and the upregulation of VEGF and VEGF receptor 2 (VEGFR2), which promoted angiogenesis and protected against neuronal apoptosis [56]. In addition, salvianolic acid B can enhance the autophagic activity of astrocytes by activating the adenosine 5′-monophosphate-activated protein kinase (AMPK)/mTOR/Unc-51-like kinase 1 (ULK1) signaling pathway and promoting the autophagic degradation of pS757-ULK1, thus playing a neuroprotective role in cerebral ischemic injury. By promoting autophagy, salvianolic acid B can remarkably increase the expression of the autophagy marker Beclin1 and the microtubule-associated protein light chain 3 B (LC3B) II/LC3B I ratio; prolong cell survival; increase the release of anti-inflammatory factors such as IL-4 and IL-10; decrease apoptosis and the levels of inflammatory factors such as interferon-gamma (IFN-γ), IL-2, and IL-6; and improve the functional status of astrocytes in the cerebral cortex of mice with oxygen–glucose deprivation. In addition, it can reduce the volume of brain infarction in mice with embolization of the left middle cerebral artery [57]. In mice with middle cerebral artery occlusion/reperfusion, salvianolic acid B, by activating astrocytic glycogenolysis to increase the degradation of accumulated glycogen, increases nicotinamide adenine dinucleotide phosphate (NADPH) and GSH levels, eliminates cellular ROS, and subsequently increases astrocyte and neuron survival and exerts neuroprotection against reperfusion injury after ischemic stroke [58].

Accumulation of α-synuclein is a key step in the pathological development of Parkinson’s disease. Chaperone-mediated autophagy is involved in the intracellular degradation of α-synuclein. Salvianolic acid B can inhibit α-synuclein aggregation in H4 human neuroglioma cells and the mouse brain. It can activate chaperone-mediated autophagy by increasing the expression of LAMP-2A (a key marker of chaperone-mediated autophagy) and macroautophagy by increasing the expression of LC3-II and LAMP-1 (a marker of lysosomal homeostasis), thereby inhibiting microglial activation and neuroinflammation [59]. In mouse models of Parkinson’s disease induced by 1-methyl-4-phenyl-1,2,3,6-tetrahydoyndine (MPTP), salvianolic acid B can alleviate the degeneration of the midbrain substantia nigra and colonic dopaminergic neurons, improve motor dysfunction and gastrointestinal dysfunction by inhibiting the colonic Toll-like receptor 4 (TLR4)/myeloid differentiation primary response protein (MyD88)/NF-κB signaling pathway, increase tyrosine hydroxylase content, and inhibit intestinal inflammatory responses by alleviating intestinal mucosal pathological damage, thereby maintaining intestinal mucosal barrier integrity. Therefore, salvianolic acid B exerts both neuroprotective and intestine-protective effects in mice with Parkinson’s disease via the intestine–brain axis [60].

The abovementioned studies suggest that salvianolic acid B protects neurons, attenuates ischemic brain injury, and acts on Parkinson’s disease. Salvianolic acid B improves detection indices mainly by exerting antioxidant and anti-inflammatory effects, increasing the expression of neurotrophic factors, reducing cell apoptosis, and promoting autophagy. It is noteworthy that salvianolic acid B exerts protective effects on both neurons and the intestine by inhibiting the TLR4/MyD88/NF-κB signaling pathway. This finding broadens the scope of research on salvianolic acid B (Figure 4 and Table 3).

Salvianolic acid B can significantly improve the abnormal physiological state and depressive-like behavior of mice with comorbid obesity and depression induced by a high-fat diet combined with chronic mild stress. In addition, it reduces the expression of CD11b (a marker of microglia activation) and directly inhibits glial cell activation in the micro-hippocampal region to alleviate neuroinflammation, thus exerting an antidepressant effect. Salvianolic acid B exerts anti-inflammatory effects by inhibiting the receptor for advanced glycosylation end products (RAGE)/Diaphanous1 (DIAPH1) pathway in microglia and it alleviates neuronal loss caused by microglia activation, thus exerting neuroprotective effects on primary microglia stimulated with palmitic acid and LPS [61]. In rats undergoing chronic mild stress, salvianolic acid B can reverse the hyperactivity of the hypothalamic–pituitary–adrenal axis and decrease the levels of inflammatory cytokines while improving the antioxidant status. Moreover, salvianolic acid B downregulates the protein expression of NLRP3 and its associated proteins ASC and cleaved caspase-1 and increases body weight and sucrose consumption rate while decreasing immobility time, thus exerting anti-depressant effects [62]. Liao et al. reported that salvianolic acid B relieved chronic mild stress-induced depressive-like state in mice by inhibiting inflammation (decreased expression of IL-6, IL-1β, TNF-α, and NF-κB p65), alleviating oxidative stress (decreased levels of 4-hydroxynonenal and MDA and increased levels of catalase (CAT), Nrf2, NQO1, and HO-1), and decreasing the expression of the ERS markers CHOP and GRP78. These effects can be attributed to the activation of the AMPK/SIRT1 signaling pathway [63].

**Figure 5:**
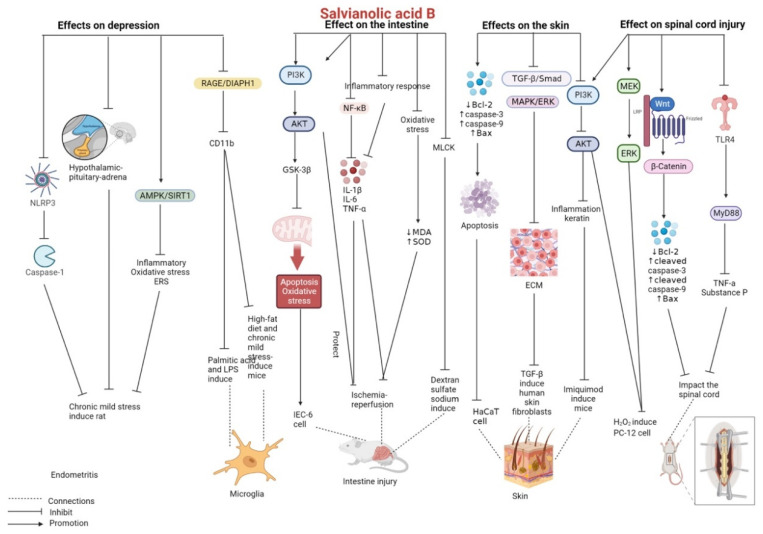
Mechanism of action of salvianolic acid B on depression, intestinal, skin, and spinal cord injury.

A few studies have reported the effects of salvianolic acid B on depression; however, its mechanism of action warrants further investigation. More in vivo and in vitro data are required to support the clinical application of salvianolic acid B (Figure 5 and Table 3).

A recent study revealed that salvianolic acid B exerts therapeutic effects against spinal cord injury [64]. Given that salvianolic acid B can upregulate miR-26a by inducing the activation of PI3K/Akt and ERK/MEK, thus alleviating H_2_O_2_-triggered injury in PC-12 cells, it may be used as a novel drug for treating spinal cord injury [65]. In rats with incomplete spinal cord injury caused by total laminectomy of the T9 vertebral plate and semicircular slice impact to the spinal cord, salvianolic acid B exerts neuroprotective effects by activating the Wnt/β-catenin signaling pathway; increasing locomotor functional recovery; decreasing the expression of Bax, cleaved caspase-3, and cleaved caspase-9 (pro-apoptotic proteins) in spinal cord tissues after injury; and increasing the expression of Bcl-2 (an antiapoptotic protein) [66]. In a study, mice’s exposed T9, T10, and T11 vertebrae were struck with a striking rod carrying a 10 g counterweight to induce spinal cord injury. Treatment of these mice with salvianolic acid B suppressed the release of TNF-a and substance P by inhibiting the activation of the TLR4/MyD88 signaling pathway, thus reducing post-injury neuropathic pain and relieving mechanical hyperalgesia in the long term [67].

The abovementioned studies indicate the great potential of salvianolic acid B in the treatment of spinal cord injury. However, the mechanism of action of salvianolic acid B should be elucidated in detail to provide valuable evidence for future clinical trials (Figure 5 and Table 3).

**Table 3:** Effects of salvianolic acid B on the cerebral nerve.

Pharmacology	Experimental Subject	Experimental Dose	Animal Experimental Drug Delivery Method	Effects	Ref.
Effects on the cerebral nerve	Oxygenose deprivation/reoxygenation of rat astrocytes	10 μMol/L	None	Decrease: ROS Increase: p-STAT3, p-AKT, BDNF, IGF1α	[54]
	Mice were infected with *Porphyromonas gingivalis*	20, 40 mg/kg	Intraperitoneal injection	Decrease: IL-1β, IL-6, Aβ, bdnf, ngf, Beta-Site APP Cleaving Enzyme 1, γ-secretase, Receptor for advanced glycation end products, ROS, MDA Increase: Recombinant A Disintegrin, Metalloprotease 10, Low-density lipoprotein receptor-related protein 1, SOD, GSH-Px	[55]
	Rats with middle cerebral artery occlusion HUVECs under hypoxia	10, 20 mg/kg 50 µg/mL	Intraperitoneal injection	Increase: VEGF, VEGFR2, STC1, mTOR, AKT	[56]
	Embolization of the left middle cerebral artery occlusion in mice Oxygen–glucose deprivation in mouse cerebral cortex astrocytes	5, 10, 20, 40, 80, 100 μg/mL 10 mg/kg	Intraperitoneal injection	Decrease: pS2448-m TOR, pS757-ULK1, IL-2, IL-6, IFN-γ, IL-10 Increase: IL-4, Beclin1, pT172-AMPK, LC3B II/LCB I	[57]
	Oxygen–glucose-deprivation/reoxygenation-induced primary astrocytes from the cortex of newborn mice Mice with middle cerebral artery occlusion/reperfusion	800 ng/mL 12 mg/kg	Intraperitoneal injection	Decrease: ROS, LDH Increase: Glucose-6-phosphate, NADPH, GSH, Glycogen phosphorylase activity	[58]
	H4 neuroglioma cells from human Bacterial Artificial Chromosome transgenic mice	50 μM 10 mg/kg	Intraperitoneal injection	Decrease: α-synuclein Increase: LAMP-1, LAMP-2A, LC3-II	[59]
	MPTP induced in mice	6.7 mL/kg	Intraperitoneal injection	Decrease: Calprotectin, TNF-α, TLR4, MyD88, NuclearNF-κB p65, p-NF-κB p65 Increase: IL-1β, Tyrosine hydroxylase, Colonic tight junction protein	[60]
	A high-fat diet combined with chronic mild stress-induced mice. Stimulation involved water and food grabbing, 45° tilt of the cage, reversal of circadian rhythm, wetting of the cage horizontal shaking, walking on ice, empty cage, flash screen, tail pinning, and restraint. Palmitic acid binding to LPS stimulates primary microglia.	10, 20, 40, 60, 80, 100, 200 μM 20 mg/kg	Intraperitoneal injection	Decrease: IL-6, IL-1β, TNF-α, iNOS, CD11b, Caspase3 Increase: NeuN+	[61]
	Rats undergoing without repeating the same stress procedure for two consecutive days, one stress/day, including with cage tilting at 45 °C, wet caging or damp bedding caging, cold water forced swimming, food deprivation, water deprivation, foot shock, tail pinching and suspension, behavior restriction, stroboscopic illumination.	20, 40 mg/kg	Intraperitoneal injection	Decrease: IL-6, IL-1β, TNF-α, MDA, ASC, cleaved caspase-1 Increase: CAT, SOD, GPx, Sucrose intake	[62]
	Rats with food deprivation followed by water deprivation, 45° cage tilting, restraint in an empty water bottle, noise, tail clamping, and damp bedding.	30 mg/kg	Intraperitoneal injection	Decrease: IL-6, IL-1β, TNF-α, NF-κB, 4-HNE, MDA, CHOP, GRP78, Microglia density of Iba-1-positive cells in the hippocampus Increase: CAT, Nrf2, NQO1, HO-1, pAMPK/AMPK, SIRT1	[63]
	H_2_O_2_-induced PC-12 cells	0, 1, 5, 10 μL	None	Decrease: Bax, cleaved caspase-3, cleaved caspase-9 Increase: Proliferating Cell Nuclear Antigen, Cyclin A, Cyclin E1, Cyclin-Dependent Kinase 2 (CDK2), Cyclin D1, CDK4, Bcl-2, miR-26a, Phosphorylation of PI3K, Phosphorylation of AKT, Phosphorylation of GSK3β	[65]
	Rats’ total laminectomy of the T9 vertebral plate. A Kirschner wire (10 g) was inserted into the aorta via a catheter with a weight that fell freely. Following this, a semicircular slice made from thin plastic was used to impact the spinal cord, and the wire was immediately removed, resulting in the incomplete injury of the rat spinal cord.	2, 10, 20 mg/kg	Intraperitoneal injection	Decrease: Bax, cleaved caspase-3, cleaved caspase-9 Increase: Bcl-2, Lymphatic enhancer factor-1 antibody, Transcription factor-1, Phosphorylation of GSK3β, β-catenin	[66]
	Mice’s exposed T9, T10, and T11 vertebrae were struck with a striking rod carrying a 10 g counterweight	30, 60 mg/kg	Gavage administration	Decrease: TLR4, MyD88, TNF-a, Substance P	[67]

### 2.4. Effects on the Heart

Salvianolic acid B has demonstrated protective effects against cardiovascular diseases. In a study, mice were administered salvianolic acid B via gavage without inducing an injury. SOD and GSH activities were increased and MDA levels were decreased in these mice, which improved antioxidant activity and increased the relative abundance of beneficial bacteria, including *Bacteroides vulgatus* and *Parabacteroides distasonis*, in mice [68]. In another study, mice were subjected to cardiac arrest induced by intravenous injection of potassium chloride, followed by cardiopulmonary resuscitation. After these mice were treated with salvianolic acid B, their myocardial performance, including cardiac output and left ventricular systolic and diastolic functions, was ameliorated within 3 h of return of spontaneous circulation. Salvianolic acid B downregulated the expression of Kelch-like ECH-associated protein 1 (Keap1) and promoted the nuclear translocation of nuclear factor erythroid-derived 2-related factor 2 (Nrf2). These changes increased the expression of the downstream antioxidant genes heme oxygenase-1 (HO-1) and NADPH:quinone oxidoreductase 1 (NQO1), inhibited cardiomyocyte apoptosis (increased expression of the antiapoptotic gene B-cell lymphoma-2 (Bcl-2) and decreased expression of the proapoptotic gene Bcl-2-associated X (Bax) and the critical mediator of apoptosis caspase-3), preserved mitochondrial morphology and function, and improved cardiac and neurological function. The antiapoptotic and antioxidant effects of salvianolic acid B were validated in H9c2 cardiomyocytes stimulated with hypoxia/reoxygenation [69].

Salvianolic acid B exerts protective effects on H_2_O_2_-induced H9c2 cardiomyocytes by activating the regulation of silent information regulator 1 (SIRT1)/AMPK/peroxisome proliferator-activated receptor-γ coactivator 1α (PGC-1α) signaling pathway, reducing ROS production, increasing mitochondrial membrane potential, inhibiting oxidative stress-induced activation of nucleotide-binding oligomerization domain-like receptor protein 3 (NLRP3) inflammasome, and decreasing the expression of the NLRP3-related proteins ASC and caspase-1. In rat models of myocardial ischemia established via ligation of the left anterior descending coronary artery, salvianolic acid B can reduce myocardial infarct size and the expression of the myocardial injury markers, including creatine kinase-MB (CK-MB) and cardiac troponin I (cTnI). In addition, it can alleviate myocardial structural abnormalities and myocardial cell apoptosis [70,71,72,73]. In H9c2 cardiomyocytes with hypoxia-induced injury, salvianolic acid B can inhibit the activation of the priming phase of NLRP3 inflammasomes to protect cardiomyocytes [74,75,76].

On the one hand, salvianolic acid B protects H9c2 cardiomyocytes against hypoxia/reoxygenation injury by inhibiting Nip3-like protein X (NIX)-mediated activation of mitochondrial autophagy, thereby increasing mitochondrial membrane potential, reducing ROS production, decreasing the protein expression of cleaved caspase-3 and LC3-II, and increasing cell viability [77]. On the other hand, salvianolic acid B downregulates the expression of maternally expressed gene 3 (MEG3), which blocks p53 and triggers AMPK activation, and decreases apoptosis, thus exerting cardioprotective effects on H9c2 cells with oxygen–glucose deprivation [78].

Salvianolic acid B reduced the damage of cardiomyocyte H9c2 glyoxylation deprivation reperfusion, improved cardiac function, reduced myocardial infarct size, decreased myocardial injury markers and inflammation-related factors, and reduced myocardial apoptosis in ischemia/reperfusion of the left anterior descending branch of the ligated coronary artery in rats by upregulating myocardial SIRT1 expression, inducing nuclear translocation, inhibiting high mobility histone box 1 (HMGB1) secretion, and inhibiting the TLR4/NF-kB signaling pathway [79]. Liu found that salvianolic acid B activated the PI3K/Akt signaling pathway to inhibit HMGB1 release, reduced the activity of the myocardial enzymes lactate dehydrogenase (LDH) and CK-MB, inhibited the release of inflammatory factors, and reduced myocardial infarct size, thereby exerting protective effects against ischemia/reperfusion injury in rat hearts [80,81]. Recently, Liu et al. reported that salvianolic acid B improved the distribution of connexin 43 (Cx43) protein in the myocardium; decreased the content of CK-MB, cTnI, LDH, MDA, ROS, and iron ions; and increased myocardial GSH levels in rat models of myocardial infarction induced via ligation of the left coronary artery. Salvianolic acid B not only reduced oxidative damage but also prevented iron-dependent cell death, thus exerting protective effects against myocardial infarction [82]. In addition, the cardioprotective effects of salvianolic acid B can be attributed to its inhibitory effects on mammalian target of rapamycin complex 1 (mTORC1)-dependent glycolysis. Salvianolic acid B decreases the abundance of pro-inflammatory M1 macrophages, increases the abundance of anti-inflammatory M2 macrophages, and decreases collagen deposition, thus alleviating cardiac dysfunction in mice with ischemia/reperfusion injury induced via ligation of the left anterior descending artery [83].

In rat models of septic myocardial injury induced via cecal ligation perforation, salvianolic acid B can reduce myocardial injury by reducing the myocardial inflammatory response and oxidative stress, inhibiting myocardial apoptosis, and increasing the expression of autophagy-related proteins [84]. In addition, salvianolic acid B protects AC16 cardiomyocytes and rats against ischemia/reperfusion injury by downregulating tripartite motif 8, upregulating glutathione peroxidase 1 (Gpx1), and alleviating apoptosis and oxidative stress [85].

Salvianolic acid B has demonstrated beneficial effects in ameliorating streptozotocin-induced myocardial fibrosis in diabetic rats. It inhibits the Ras homolog gene family member A (RhoA)/Rho-associated protein kinase (ROCK1) signaling pathway and downregulates the protein expression of α-SMA, collagen I, and collagen III to exert protective effects against cardiovascular disease caused by diabetes [86]. Salvianolic acid B can enhance the DNA methylation of the insulin-like growth factor-binding protein 3 (IGFBP3) promoter and induce the nuclear translocation of IGFBP3 in HUVECs under hypoxia. In addition, it suppresses IGFBP3 to increase the expression of VEGFR2 and VEGFA, promotes angiogenesis both in vivo and in vitro, and attenuates myocardial remodeling, cardiac dysfunction, and myocardial fibrosis in diabetic mice induced by streptozotocin [87]. In high-glucose-stimulated cardiac fibroblasts (CFs) of rats, salvianolic acid B can inhibit cell proliferation and trans-differentiation by inhibiting the Wnt/β-catenin signaling pathway and decreasing the expression of p-GSK 3β and α-SMA [88,89]. Zhao et al. established rat models of diabetes mellitus through the administration of a high-sugar and high-fat diet combined with streptozotocin injection. Treatment of these rats with salvianolic acid B downregulated Bax and caspase-3 expression and upregulated Bcl-2 expression, eventually reducing serum CK-MB levels and inhibiting cardiomyocyte apoptosis in diabetic rats [90]. Ultra-performance liquid chromatography with mass spectrometry (UPLC/MS) revealed that salvianolic acid B exerted protective effects against coronary heart disease in rats (fed a high-fat diet combined with injection of vitamin D3) by regulating glycerophospholipid, sphingolipid, and arachidonic acid metabolism and alleviating oxidative-stress-induced damage and lipid peroxidation [91].

Salvianolic acid B can induce the activation of the Nrf2/ARE signaling pathway to achieve antioxidant effects when H_2_O_2_ interferes with rat bone marrow mesenchymal stem cells (BMSCs). It inhibits the apoptosis of BMSCs by upregulating the Bcl-2/Bax ratio in the presence of oxidative damage. In addition, it remarkably increases the expression of the myocardial differentiation markers GATA4 and cTnT, thus promoting the differentiation of BMSCs into cardiomyocytes [92]. Zhao et al. demonstrated that salvianolic acid B inhibited angiotensin (Ang II)-induced proliferation of vascular smooth muscle cells in vitro and alleviated intimal hyperplasia in mice with carotid artery ligation by downregulating the expression of microRNA-146a (miR-146a) and the positive regulators of the cell cycle Krüppel-like factor 5 (KLF5) and cyclin D1, thus exerting cardioprotective effects [93]. Furthermore, salvianolic acid B can suppress endoplasmic reticulum stress (ERS) and downregulate TXNIP (a connecting ERS and inflammation critical function) by inhibiting the AMPK/Forkhead box O4/KLF2 and Syndecan-4/Rac1/activating transcription factor 2 signaling pathway. It substantially prevents bone-marrow-derived endothelial progenitor cell damage associated with ERS by decreasing intracellular ROS levels, inducing NLRP3-dependent pyroptosis, and increasing HO-1 and SOD2 levels, eventually attenuating ERS-induced endothelial injury. Therefore, salvianolic acid B is a potential candidate drug for the treatment of atherosclerotic heart disease [94].

**Figure 6:**
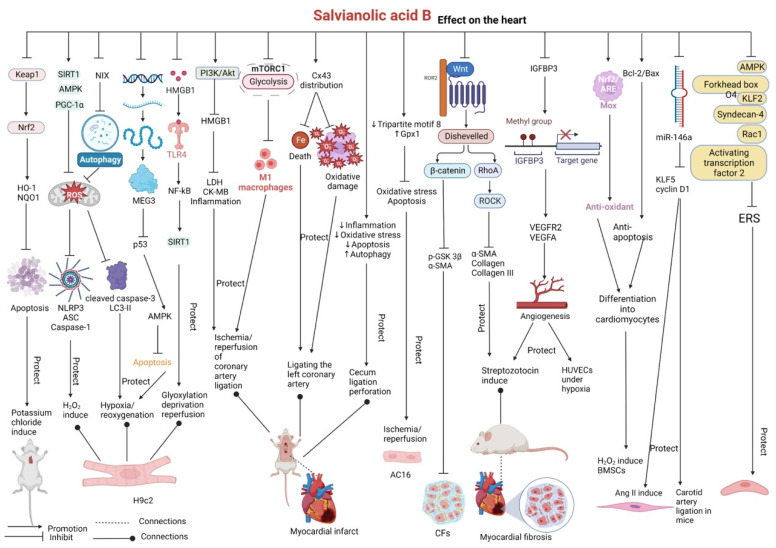
Mechanism of action of salvianolic acid B’s effects on the heart.

To date, studies have reported the use of salvianolic acid B in the treatment of heart diseases such as cardiac arrest, myocardial ischemia, cardiomyocyte injury, myocardial infarction, cardiac dysfunction, and myocardial fibrosis. Salvianolic acid B has demonstrated cardioprotective effects in both in vitro and in vivo models of heart diseases induced using the following methods: hypoxia/reoxygenation, H_2_O_2_ stimulation, hypoxia, oxygen–glucose deprivation, glyoxylation deprivation reperfusion (H9c2 cardiomyocytes), ischemia/reperfusion (AC16 cells), Ang II stimulation (vascular smooth muscle cells), potassium injection (mice), carotid artery ligation (mice), ischemia/reperfusion injury induced via coronary artery ligation, cecal ligation perforation, streptozotocin stimulation, high-glucose stimulation, and administration of high-sugar and high-fat diet combined with streptozotocin injection (rats). In addition, rat BMSCs are commonly used in studies on salvianolic acid B. Altogether, salvianolic acid B plays a protective role in cardiovascular diseases by exerting antioxidant, antiapoptotic, antifibrotic, and anti-inflammatory effects; regulating autophagy and cell cycle regulators; preventing iron-dependent cell death; promoting angiogenesis; and suppressing ERS (Figure 6 and Table 4).

### 2.5. Effects on Blood Vessels

In a study on zebrafish models of vascular injury induced by the vasopressor PTK787, virtual screening and activity evaluation revealed that salvianolic acid B played a key role in promoting angiogenesis [95]. Niu et al. reported that salvianolic acid B promoted angiogenesis, upregulated the expression of M2 marker genes, and reduced muscle edema by enhancing macrophage polarization via the SIRT1/PI3K/Akt pathway in mice with hind limb ischemia. In addition, cell migration and tube formation were promoted in endothelial cells stimulated with culture supernatant derived from salvianolic-acid-B-treated macrophages, resulting in the alleviation of peripheral arterial disease [96].

In a study on HUVECs stimulated with high concentrations of glucose and carbonyl cyanide m-chlorophenyl hydrazone and C57BL/KsJ db/db mice, salvianolic acid B increased Bcl-2 expression and reduced Bax, Beclin1, Parkin, and PTEN-induced kinase 1 (Pink1) expression, thereby protecting endothelial cells from apoptosis and mitophagy. Salvianolic acid B markedly enhanced cell migration, mitochondrial activity, and intracellular Ca2+ levels in HUVECs in vitro. In addition, it ameliorated hyperlipidemia, hyperglycemia, hyperinsulinemia, insulin resistance, and diabetes-induced vascular endothelial dysfunction in db/db mice [97]. In Ang II-induced HUVECs, salvianolic acid B can activate downstream pathways by enhancing the activation of the PI3K/Akt and MAPK (extracellular signal-regulated protein kinases 1/2 (ERK1/2), p38, and c-Jun N-terminal kinase (JNK)) signaling pathways. In addition, it can alleviate endothelial cell damage by activating the Nrf2/HO-1 signaling pathway while decreasing ROS and MDA levels and increasing NO levels, thus acting as an antioxidant [98].

The lack of the bridging molecule MFGE8 in bone-marrow-derived cells increases the accumulation of apoptotic cells in atherosclerotic plaques and accelerates plaque development [99,100,101]. In a study, low-density lipoprotein receptor gene knockout (LDLR-/-) mice were fed high-fat diets to induce atherosclerosis, and oxidized low-density lipoprotein (ox-LDL) was used to induce cytosolic burial in RAW264.7 mouse macrophages. Treatment with salvianolic acid B upregulated the expression of the TAM tyrosine kinase receptor family (TYRO3, AXL, and MERTK) and MFGE8 and promoted efferocytosis in both mice and mouse macrophages. In addition, it remarkably reduced total cholesterol (TC), triglyceride (TG), and low-density lipoprotein–cholesterol (LDL-c) levels in mice, thus exerting anti-atherosclerotic effects [102]. Salvianolic acid B can ameliorate atherosclerosis in LDLR-/- mice and alleviate LPS-induced inflammation in RAW264.7 cells by inhibiting the phosphorylation of STAT3/NF-κB. It decreases levels of mice blood lipids TC, TG, and LDL-C and downregulates the expression of the inflammatory factors IL-1β, IL-6, and TNF-α [103]. Salvianolic acid B can alleviate atherosclerosis by promoting anti-inflammation, with the MAPK/NF-κB signaling pathway playing an important role in this protective effect. It can inhibit the MAPK/NF-κB signaling pathway; decrease the levels of IL-1β, IL-6, and TNF-α; downregulate the protein expression of vascular cell adhesion molecule (VCAM) and inducible nitric oxide synthase (iNOS); and reduce the phosphorylation of JNK, p38, ERK1/2, and IκB proteins in LDLR-knockout mice fed a high-fat diet and in ox-LDL-induced or LPS-induced RAW264.7 cells [104]. Salvianolic acid B can inhibit the Yes-associated protein (YAP)/transcriptional coactivator with the PDZ-binding motif (TAZ)/JNK signaling pathway to attenuate the development of atherosclerosis in ApoE-/- mice fed a high-fat diet. It decreases the expression of the inflammation-related proteins JNK, NF-κB, and TNF-α in ox-LDL-induced ECs and pericytes in vitro. It decreases the expression of the inflammation-related factors IL-6, IL-1β, and TNF-α and reduces aortic root sinus lesion size. In addition, it decreases ROS, MDA, and Annexin V (apoptosis marker) levels and increases SOD and GSH-PX activity, thus alleviating oxidative stress and inhibiting apoptosis to delay atherogenesis [105].

In RAW264.7 macrophages, salvianolic acid B can inhibit the activation of the NF-κB signaling pathway; downregulate Akt/mTOR signaling; inhibit M1 macrophage polarization; promote M2 macrophage polarization; decrease the expression of the M1 markers iNOS, TNF-α, and IL-6l; and increase the expression of the M2 markers Arg-1 and IL-10, thereby promoting autophagy and exerting anti-atherosclerotic effects [106]. Salvianolic acid B not only enhances RAW264.7 macrophage autophagy by inhibiting the Akt/mTOR signaling pathway to attenuate macrophage apoptosis but also inhibits cholesterol-crystal-induced release of the pro-inflammatory cytokines TNF-α and IL-6, exerting a beneficial effect on atherosclerosis [107]. Gao et al. demonstrated that salvianolic acid B upregulated AMPK phosphorylation, downregulated mTOR signaling, and promoted autophagy to protect HUVECs from apoptosis under oxidative stress, thus increasing LC3-II and Beclin-1 expression and reducing p62, cytochrome c, and caspase-3 expression. These findings suggest a novel approach to the treatment of atherosclerosis [108].

On the one hand, salvianolic acid B inhibits Ang II-induced migration and transformation of rat extravascular membrane fibroblasts and reduces ROS and α-SAM levels [109]. On the other hand, salvianolic acid B inhibits high-phosphorus-induced calcification of VSMCs and their transformation to the osteogenic phenotype by activating autophagy, resulting in a decrease in the expression of runt-related transcription factor-2 (Runx2) and osteopontin (OPN) (molecular markers of osteogenic differentiation of VSMCs) and an increase in the expression of Beclin-1, LC3-I/II (markers of autophagy), calponin, and SM22 (markers of the shrinkage phenotype of VSMCs) [110].

Salvianolic acid B has remarkable anti-thrombosis effects [111]. In a study, the combined use of molecular docking and zebrafish models revealed that salvianolic acid B attenuated zebrafish caudal vein thrombosis and promoted blood circulation. Zheng et al. [112] demonstrated that salvianolic acid B markedly decreased the likelihood of thrombosis by inhibiting the NF-κB/JNK/p38 MAPK signaling pathway in TNF-α-stimulated human EA.hy926 cells. In addition, it prolonged prothrombin time and activated partial thromboplastin time, decreased fibrinogen concentration, and inhibited platelet aggregation induced by adenosine diphosphate in rat blood [113].

**Figure 7:**
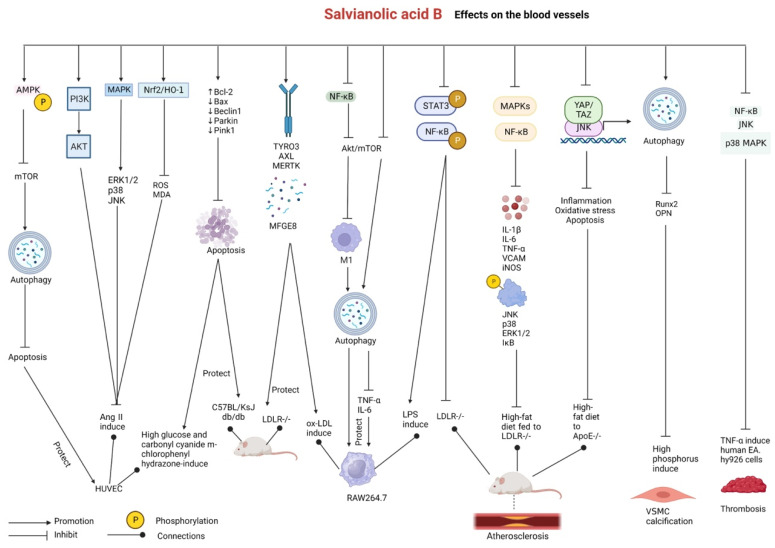
Mechanism of action of salvianolic acid B on blood vessels.

Danshen is traditionally used to treat blood vessel diseases, as it promotes blood circulation and dispels hemostasis. The abovementioned studies validate that salvianolic acid B derived from Danshen promotes angiogenesis; alleviates vascular endothelial dysfunction, inhibits atherosclerosis, VSMC calcification, and thrombosis; and inhibits the activity of extravascular membrane fibroblasts both in vitro and in vivo. These studies provide substantial evidence of the traditional use of salvianolic acid B. The protective effects of salvianolic acid B on the blood vessel system are a core focus of research. Salvianolic acid B plays a therapeutic role in blood vessel diseases by exerting antioxidant and antiapoptotic effects by improving the activity of antioxidant enzymes (SOD, NO, and GSH-Px) and regulating the expression of apoptosis-related proteins. In addition, these protective effects can be achieved through a reduction in cell adhesion (Figure 7 and Table 5).

### 2.6. Effects on the Kidney

In iopromide-induced HK-2 human renal cortical proximal tubular epithelial cells, salvianolic acid B can inhibit the release of pro-inflammatory cytokines including IL-1β, IL-18, TNF-α, apoptosis-associated speck-like protein containing a CARD (ASC), caspase-1, and NF-κB by suppressing the TLR4/NF-κB/NLRP3 signaling pathway. In addition, it inhibits apoptosis and ROS production and enhances cellular mitochondrial membrane potential. Salvianolic acid B at a concentration of 100 μMol/L can alleviate ERS and cell damage, increase cell viability, and reduce the expression of ERS-related proteins, including 78-kDa glucose-regulated protein (GRP78), p-eIF2α, p-JNK, and C/EBP-homologous protein (CHOP) [114,115,116]. Gao et al. reported that salvianolic acid B effectively increased cell viability, upregulated the expression of Bcl-2 (antiapoptotic protein), downregulated the expression of Bax and cleaved caspase-3 (pro-apoptotic protein), inhibited apoptosis by alleviating oxidative stress, reduced ROS levels, activated the Akt signaling pathway, and inhibited the ERK signaling pathway in iopromide-treated HK-2 cells [117]. Pang et al. used the modified chronic serum disease method to replicate the mesangial proliferative glomerulonephritis rat model, in which salvianolic acid B inhibited glomerular cell apoptosis and reduced inflammatory damage by reducing NF-κB-p65/Bax and TNF-α activity and inhibiting caspase-3-dependent apoptosis and the release of cytosolic inflammatory factors [118]. In rats with cationic calf serum albumin-induced chronic glomerulonephritis, salvianolic acid B can improve renal function by inhibiting inflammation (decreased expression of IL-1β and IL-6), alleviating oxidative stress (decreased MDA levels and increased SOD and GSH activities), and promoting autophagy (decreased p62 expression and increased the LC3 II/LC3 I ratio and Beclin1 expression) [119]. Salvianolic acid B significantly ameliorated kidney function and pathological changes in rats with cationic bovine serum albumin-induced membranous nephropathy and LPS-induced human mesangial cells by upregulating miR-145-5p to inhibit the PI3K/Akt signaling pathway and by upregulating mesangial cell autophagy, thus reducing cell proliferation and inflammation [120].

In a recent study, interstitial fibrosis was induced in the rat kidney via unilateral ureteral obstruction. Treatment of these rats with salvianolic acid B inhibited iron-dependent death by activating the Nrf2/Gpx4 pathway, decreased MDA content, increased SOD activity, and reduced oxidative stress, resulting in an improvement in interstitial fibrosis [121]. Renal tubular EMT promotes the progression of renal interstitial fibrosis. In high-glucose-stimulated NRK-52E rat proximal renal tubular epithelial cells, salvianolic acid B upregulates phosphatase and tensin homology deleted on chromosome 10 (PTEN) expression by activating peroxisome proliferator-activated receptor gamma (PPARγ), which in turn inhibits the fibrogenic effects of the PI3K/Akt signaling pathway and eventually inhibits EMT [122]. In rats subjected to unilateral nephrectomy, salvianolic acid B can reduce the expression of EMT-related proteins, including fibronectin, α-SMA, and TGF-β; activate autophagy; and upregulate the expression of Sirt1, thereby alleviating kidney dysfunction. In TGF-β1-induced HK-2 human kidney proximal tubular epithelial cells, salvianolic acid B can reverse EMT during renal fibrosis by activating Sirt1-mediated autophagy [123].

In a study, mouse models of ischemia/reperfusion-induced acute kidney injury were established via unilateral nephrectomy contralateral to renal artery clamping of the renal tip, whereas HK-2 cells were subjected to hypoxia–reoxygenation injury. Treatment with salvianolic acid B inhibited the activation of NLRP3 by promoting the nuclear expression of Nrf2 and activating the Keapl–Nrf2/HO-1 antioxidant pathway, thereby suppressing caspase-1-mediated cell scorching, secretion of inflammatory factors, and oxidative stress and effectively improving acute kidney injury [124]. In mouse models of left ureteral ligation, salvianolic acid B can decrease the expression of fibroblast growth factor-2 (FGF-2), serum creatinine, blood urea nitrogen, and TGF-β1. In Ang II-stimulated HK-2 cells, salvianolic acid B can downregulate α-SMA expression and upregulate syndecan-1 (SDC1)/E-cadherin expression. Salvianolic acid B exerts renal-protective effects by inhibiting the heparanase (HPSE)/SDC1 axis both in vivo and in vitro [125].

In rats with unilateral ureteral obstruction, salvianolic acid B improves kidney dysfunction; increases the expression of PAR-3 polyclonal antibody (Par-3); and reduces the expression of connective tissue growth factor (CTGF), platelet-derived growth factor C (PDGF-C), and PDGFR-α. In HK-2 cells stimulated with human serum albumin, salvianolic acid B can protect against fibrosis by inhibiting the PDGF-C/PDGFR-α signaling pathway while inhibiting apoptosis and ERS [126]. Salvianolic acid B remarkably suppresses apoptosis (Bax and cleaved caspase-3) and alleviates ERS (BIP, P-eIF2α, ATF4, CHOP, ATF6, IRE1α, and XBP1s) in the kidney of mice fed a high-fat diet, thus suppressing the progression of kidney injury. In addition, salvianolic acid B can attenuate the adverse effects (such as apoptosis and ERS) of palmitic acid in HK-2 cells [127].

Salvianolic acid B ameliorates renal fibrosis and inflammatory responses and improves the hyperlipidemic status by inhibiting the TGF-β1/Smad and NF-κB signaling pathways in db/db mice with high-fat-diet-induced diabetic nephropathy [128]. It can inhibit oxidative stress and reduce extracellular matrix (ECM) secretion by inhibiting the TGF-β1/Smad and TGF-β1/p38MAPK signaling pathways, thereby improving renal function and fibrosis in rats with diabetic nephropathy induced by high-glucose and high-fat diet combined with streptozotocin administration. In addition, salvianolic acid B can inhibit high-glucose-induced differentiation of human glomerular mesangial cells into myofibroblasts and decrease the secretion of collagen type I, collagen type III, fibronectin, and laminin [129,130]. In a study, metabolomic analysis via UPLC-Q-TOF-MS revealed that salvianolic acid B interfered with metabolic disorders in db/db mice with diabetic nephropathy by regulating lipid and lipoprotein metabolism, biosynthesis of unsaturated fatty acids, arachidonic acid metabolism, sphingolipid metabolism, acetylcholine synthesis, fatty acid metabolism, triacylglycerol metabolism, ketone body metabolism, and other glucolipid metabolic pathways [131].

**Figure 8:**
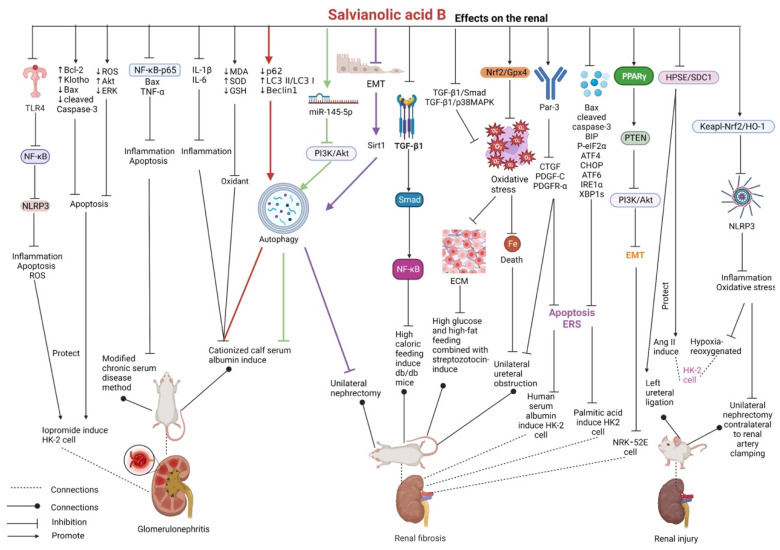
Mechanism of action of salvianolic acid B on kidneys.

The abovementioned studies validate the therapeutic effects of salvianolic acid B against various renal diseases, including glomerulonephritis, renal function injury, renal fibrosis, and diabetic nephropathy. Salvianolic acid B exerts renal-protective effects by inhibiting the release of inflammatory cytokines, alleviating oxidative stress and ERS, inhibiting apoptosis and EMT, and promoting autophagy. In addition, NF-κB, NLRP3, Nrf2, TGF-β1, and Smad can be considered the targets of salvianolic acid B for the treatment of renal diseases. However, further studies are warranted to validate these targets (Figure 8 and Table 6).

### 2.7. Effects on the Intestine

Salvianolic acid B maintains mitochondrial membrane potential; protects mitochondrial function; reduces apoptosis; decreases caspase-3, Cyt-c, Bax, and ROS levels; and elevates Bcl-2 levels by activating the PI3K/Akt/GSK-3β pathway, which protects IEC-6 rat intestinal epithelial cells from oxidative stress. It remarkably alleviates jejunum and ileum injuries in rats with occlusion of the superior mesenteric artery followed by reperfusion [132,133]. In a study, the superior mesenteric artery of rats was clamped to establish models of intestinal ischemia/reperfusion injury. Treatment of these rats with salvianolic acid B inhibited the expression of IL-1β, IL-6, and TNF-α (inflammatory factors) by inhibiting the NF-κB signaling pathway and alleviated intestinal ischemia/reperfusion injury by activating the PI3K/Akt signaling pathway [134]. In addition, salvianolic acid B exerted protective effects on the intestinal mucosa by reducing oxidative stress (decreased MDA activity and elevated SOD activity) and improving intestinal permeability [135]. Salvianolic acid B can decrease the expression of myosin light chain kinase (MLCK), a marker of tight junction dysfunction, and reverse tight junction barrier dysfunction to protect mice with colitis induced by dextran sulfate sodium in drinking water [136].

Salvianolic acid B has certain therapeutic effects against intestinal diseases. Several in vitro and in vivo studies have demonstrated that salvianolic acid B can effectively protect against intestinal ischemia/reperfusion injury and colitis mainly by alleviating oxidative stress and inhibiting inflammatory responses (Figure 5 and Table 7).

### 2.8. Effects on the Bone

In a study, nucleus pulposus cells extracted from rat caudal intervertebral discs were treated with H_2_O_2_ in vitro, and degeneration was induced in caudal intervertebral discs in rats through percutaneous needling. Treatment with salvianolic acid B activated the JAK2/STAT3 signaling pathway, thereby inhibiting oxidative stress in myeloid cells (elevated levels of SOD, GSH-Px, and CAT and decreased levels of MDA and ROS), apoptosis (decreased Bax and cleaved caspase 3 levels and elevated Bcl-levels), and inflammatory responses (decreased IL-1β, IL-6, and TNF-α expression). In addition, it reduced the expression of MMPs and A disintegrin-like and metalloproteinase with thrombospondin motifs (ADAMTS), elevated the expression of collagen II and aggrecan, and inhibited ECM degradation, eventually alleviating disc degeneration [137,138]. Yan et al. induced intervertebral disc degeneration in New Zealand white rabbits via nucleus pulposus aspiration. Mesenchymal stem cell transplantation combined with salvianolic acid B treatment was more effective in repairing degenerated intervertebral discs than stem cell transplantation alone [139].

Salvianolic acid B plays a key role in accelerating bone remodeling, which can initiate vascularization and accelerate the formation of new bone after rapid maxillary expansion in rats, accompanied by an increase in the newly formed bone area and the number of capillaries, osteoblasts, and osteoclasts and a decrease of inflammatory cells [140]. Salvianolic acid B can promote osteogenic differentiation of human BMSCs in vitro by promoting the expression of the long noncoding RNA (lncRNA) metastasis-associated lung adenocarcinoma transcript 1 (MALAT1). In a study on rat models of osteoporotic fracture caused by bilateral ovarian removal and sawing of the femur, 3D imaging and evaluation of bone density revealed that salvianolic acid B accelerated the healing of osteoporotic fractures by promoting angiogenesis [141]. A high concentration of dexamethasone can inhibit osteogenesis and induce mitochondrial dynamic disorder in BMSCs, whereas 5 μM of salvianolic acid B can effectively inhibit mitochondrial production and improve osteogenesis by scavenging intracellular ROS [142]. Salvianolic acid B can significantly increase cell viability and stimulate bone formation by activating the PI3K/Akt signaling pathway and promoting the expression of alkaline phosphatase (ALP), OPN, collagen I, and osteocalcin (OCN) in MC3T3-E1 mouse osteoblasts [143]. In rat skull osteoblasts stimulated with prednisolone acetate, salvianolic acid B exerts protective effects by increasing the expression of Nrf2, HO-I, Runx2, and the downstream genes of Runx2 that are involved in differentiation and bone formation, including Osx, OCN, IGF-I, and collagen I [144].

Salvianolic acid B promotes osteogenesis and has been used in studies employing gingival mesenchymal stem cells and periodontal ligament stem cells (PDLSCs). It can enhance the proliferative, migratory, and invasive capabilities of human gingival mesenchymal stem cells by activating the PI3K/Akt pathway. In addition, it can enhance osteogenic differentiation; increase the expression of osteogenic proteins such as Runx2, OCN, collagen I, and ALP; inhibit lipogenic differentiation; and decrease the expression of lipogenic proteins such as PPARγ and C/EBPα [145]. In a study, PDLSCs obtained from orthodontic patients were stimulated with H_2_O_2_. Treatment of these cells with salvianolic acid B enhanced cellular antioxidant capacity by activating Akt/Nrf2 signaling, reducing ROS levels, and promoting SOD and GSH activities, thereby promoting osteogenic differentiation of PDLSCs (increased mRNA expression of ALP, collagen I, Runx2, and OCN), inhibiting lipogenic differentiation of PDLSCs (decreased mRNA expression of PPARγ, LPL, and C/EBPα), and eventually increasing PDLSC viability [146]. Salvianolic acid B promotes the differentiation of human PDLSCs into osteoblasts by activating the Wnt/β-catenin signaling pathway, thereby increasing the expression of osteogenesis-associated genes such as Runx2, BMP2, OSX, and OCN in PDLSCs [147].

Salvianolic acid B can alleviate osteoarthritis development by reducing body weight and decreasing inflammatory responses in mouse models of osteoarthritis induced by a high-fat diet and anterior cruciate ligament transection/medial meniscus removal. In a study on palmitic acid-stimulated ATDC5 mouse chondrocytes, salvianolic acid B was found to protect against inflammatory and apoptotic injury by upregulating the expression of the lncRNA KCNQ1 overlapping transcript 1 and activating autophagy via the miR-128-3p/SIRT1/JAK2/STAT3 pathway [148].

**Figure 9:**
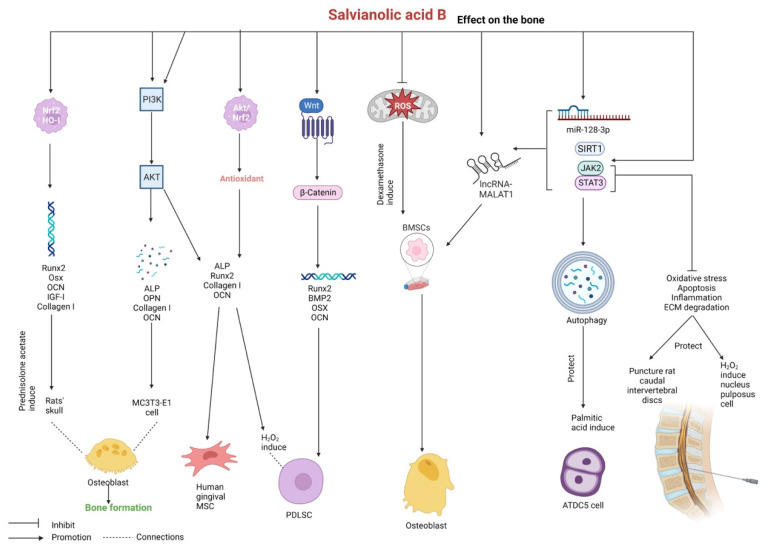
Mechanism of action of salvianolic acid B on bone.

The abovementioned studies suggest that salvianolic acid B can effectively treat bone-related diseases by inhibiting intervertebral disc degeneration, accelerating bone formation, and alleviating osteoarthritis. Salvianolic acid B exerts protective effects against bone-related diseases mainly by regulating the JAK2/STAT3, lncRNA-MALAT1, PI3K/Akt, and miR-128-3p/SIRT1/JAK2/STAT3 signaling pathways. Moreover, different bone-related disease models have different characteristics. The treatment effects of salvianolic acid B have been verified and discussed in various bone-related disease models. In particular, salvianolic acid B has been demonstrated to accelerate bone formation in human gingival mesenchymal stem cells and PDLSCs. This finding broadens the scope of research on salvianolic acid B and significantly contributes to its development (Figure 9 and Table 8).

### 2.9. Effects on the Liver

In rat models of alcoholic liquid-diet-induced liver injury, salvianolic acid B can remarkably upregulate the phosphorylation of AMPKα1 and mediate the activation of SIRT1 to restore ethanol dehydrogenase activity and promote alcohol metabolism, eventually protecting against alcoholic liver injury [149]. In addition, salvianolic acid B exerts protective effects against chronic alcoholic liver disease by improving the survival of ethanol-stimulated HepG2 cells by regulating the AMPKα1/SIRT1 pathway [150]. In a study on ApoE-knockout mice with non-alcoholic fatty liver disease induced by a high-fat diet, salvianolic acid B was found to enhance hepatic autophagy (elevated Beclin1, p62, and LC3 expression) by activating the AMPK pathway, attenuate hepatic oxidative stress (decreased Nrf2 and HO-1 expression), and reduce hepatic inflammation (decreased NF-κB, IL-6, and TNF-α expression), thus reducing liver damage and protecting the liver [151].

Salvianolic acid B protects against arsenic trioxide-induced injury in HepaRG human hepatocytes by maintaining mitochondrial function, elevating the Bcl-2/Bax ratio, and upregulating the cell survival protease p-Akt, thereby inhibiting hepatocyte apoptosis [152]. In rat models of arsenic trioxide-induced liver injury, salvianolic acid B can remarkably improve inflammation or necrosis in the liver tissue by elevating SOD levels, decreasing MDA levels, and alleviating oxidative stress [153]. In some studies, CCl_4_ has been used to induce acute and chronic liver injuries in mice, whereas H_2_O_2_ has been used to induce oxidative damage in the rat hepatic stellate cell (HSC) line HSC-T6. Treatment with salvianolic acid B inhibits the phosphorylation of Smad2 both at the C-terminus and link region via the TGF-β/Smad signaling pathway, improves the histological characteristics of the liver, decreases the levels of aspartate aminotransaminase (AST) and alanine aminotransferase (ALT), and attenuates oxidative stress (increased SOD levels and decreased GSH levels) to protect against acute and chronic liver injury. In addition, upregulation of the Nrf2/HO-1 signaling pathway by salvianolic acid B plays a key role in suppressing CCl_4_-induced acute liver injury [154,155]. In mouse models of senecionine-induced acute liver injury, salvianolic acid B can regulate oxidative stress (increased SOD levels and decreased GSH levels) and inhibit liver fibrosis-related factors, including MMP-9, TGF-β1, and phosphorylated STAT3, by modulating the coagulation system (decreased expression of plasminogen activator inhibitor 1 (PAI-1)). In addition, salvianolic acid B alleviates liver injury in mice by decreasing ALT and AST levels, improving pathological conditions such as intrahepatic sinusoidal hemorrhage and hepatocyte necrosis, and reducing the levels of serum pyrrolizidine alkaloids, which are the markers of toxicity of pyrrole–protein adducts [156]. In rat models of cholestatic liver injury induced by α-naphthyl isothiocyanate, salvianolic acid B alleviates liver damage by regulating bile acid transporters and metabolic enzymes such as Bsep, Mrp3, Oatp2, Cyp3a2, and Ugt1a1 to promote bile acid metabolism. In addition, it reduces inflammation by inhibiting the NF-κB/IκB, p38 MAPK, and JNK MAPK inflammatory signaling pathways, thereby improving liver function and alleviating cholestatic liver injury [157,158]. In mouse models of cecal ligation- and puncture-induced septic liver injury, salvianolic acid B exerts protective effects by activating SIRT1/PGC-1α signaling, improving pathological characteristics, reducing serum AST and ALT levels, and inhibiting hepatic apoptosis (decreased Bax and increased expression of Bcl-2), thus decreasing the expression of the inflammatory cytokines TNF-α and IL-6 [159]. Salvianolic acid B improves the growth performance of Nile tilapia with cyclophosphamide-induced liver injury and alleviates liver lesions by regulating the expression of genes involved in the antioxidant (Nrf2), anti-autophagy (AMPK/mTOR), and antiapoptotic (MAPK) pathways [160].

In mouse models of diethylnitrosamine-induced hepatic fibrosis and HSC-T6 stimulated with TGF-β1, salvianolic acid B can remarkably promote the apoptosis of hepatic fibrotic cells by upregulating the expression of cleaved caspase-9 [161,162]. Salvianolic acid B can attenuate liver fibrosis by inhibiting the TGF-β/Smad and MAPK pathways, especially by inhibiting MAPK-mediated P-Smad2/3L signaling. Salvianolic acid B alleviates diethylnitrosamine-induced liver fibrosis in mice by decreasing the protein expression of hepatic fibrosis-related markers (α-SMA, collagen I, and TGF-β1) and improving histopathological characteristics. In addition, it impedes cell migration and collagen I production in TGF-β1-induced HSC-T6 and LX-2 human HSCs [163]. In rat models of carbon-tetrachloride-induced liver fibrosis, salvianolic acid B exerts anti-fibrotic effects by downregulating TGF-β1, inhibiting the hedgehog signaling pathway, and reducing the expression of hedgehog signaling pathway-related genes, including Sonic hedgehog (Shh), membrane protein receptor protein patched homolog 1 (Ptch1), membrane protein receptor smoothened (Smo), and transcription factor glioma-associated oncogene homolog 1 (Gli1), thereby inhibiting the activation of HSCs, which are key cells involved in the development of a fibrotic environment [164]. Salvianolic acid B can prevent the proliferation and activation of LPS-induced LX-2 cells by enhancing the antifibrotic fibroblast growth factor (FGF19)/FGFR4 signaling pathway [165]. In TGF-β1-induced JS1 mouse immortalized stellate cells and LX2 cells, salvianolic acid B can inhibit activation and autophagy by downregulating the ERK, p38, and JNK pathways and decreasing the expression of proteins related to autophagy and liver fibrosis [166]. Zhang et al. established rat models of liver fibrosis induced by dimethylnitrosamine for in vivo analysis and used TGF-β1-stimulated HSCs for in vitro analysis. Treatment with salvianolic acid B inhibited the activity of myocyte enhancer factor 2 (MEF2) and reduced the expression of the HSC activation markers α-SMA and collagen I, thus exerting anti-fibrotic effects [167].

**Figure 10:**
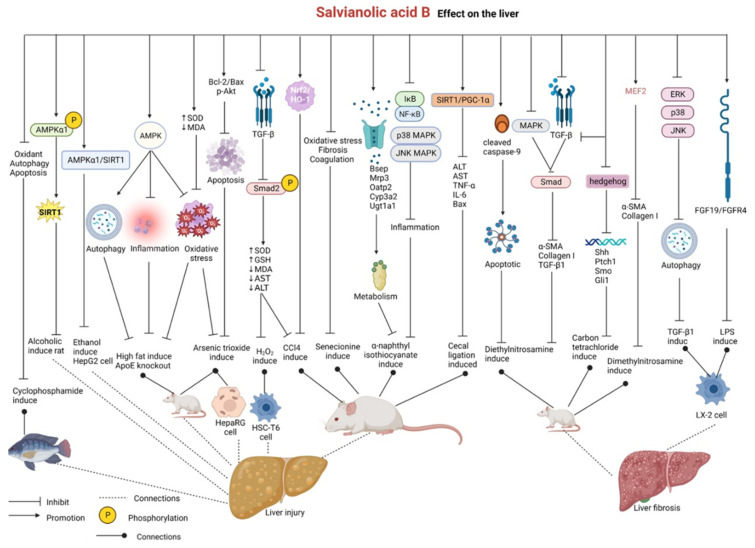
Mechanism of action of salvianolic acid B on the liver.

Altogether, salvianolic acid B can be used in the treatment of chronic or acute liver injury, non-alcoholic fatty liver disease, alcoholic liver injury, and liver fibrosis. Remarkably, the multiple bioactivities of salvianolic acid B are closely associated with its ability to activate the AMPK, SIRT1, Nrf2/HO-1, and FGF19/FGFR4 signaling pathways; inhibit the TGF-β/Smad, MAPK, and NF-κB/IκB signaling pathways; regulate oxidative stress; and inhibit apoptosis, inflammation, and the expression of liver fibrosis-related factors. These data provide a certain pharmacological basis for the development and use of salvianolic acid B in treating liver diseases and may help expand the scope of research on new drugs for clinical application (Figure 10 and Table 9).

### 2.10. Effects on the Skin

Salvianolic acid B plays a therapeutic role in skin warts by increasing caspase-3, caspase-9, and Bax expression and decreasing Bcl-2 expression, thus inducing HaCaT human immortalized cells apoptosis and blocking cells from staying in the G0/G1 phase [168]. In mouse models of bleomycin-induced systemic sclerosis, salvianolic acid B alleviated skin fibrosis by reducing skin thickness and collagen deposition. In human skin fibroblasts stimulated with TGF-β, salvianolic acid B suppressed the expression of ECM-related genes and cell proliferation by inhibiting the TGF-β/Smad and MAPK/ERK signaling pathways. Salvianolic acid B exerts anti-fibrotic effects by inhibiting oxidative stress, the cell cycle, and the p53 signaling pathway [169]. Salvianolic acid B inhibits the PI3K/Akt signaling pathway, thus inhibiting psoriatic inflammation (downregulation of IL-22, IL-23, IL-17A, IL-1β, and IL-6) and the expression of keratin markers (downregulation of K16 and K17) in mouse models of psoriasis induced by imiquimod [170].

To date, studies have reported the use of salvianolic acid B in the treatment of skin diseases including skin warts, skin fibrosis, and psoriasis both in vitro and in vivo, and its mechanism of action has been investigated as well. The findings of these studies indicate the potential applicability of salvianolic acid B in the treatment of skin diseases and its diverse therapeutic roles. Further studies are warranted to validate these findings (Figure 5 and Table 10).

### 2.11. Other Effects

In addition to exerting the therapeutic effects mentioned above, salvianolic acid B can protect the retina and airway; promote fat graft survival; protect the testicles; improve crush syndrome; prevent cystine stones; alleviate hand, foot, and mouth disease; inhibit obesity; enhance the immune system; accelerate wound healing; and act as an anti-convulsant. Some of these effects are briefly discussed below (Table 11).

In high-glucose-stimulated primary Müller cells from the rat retina, salvianolic acid B can inhibit apoptosis by significantly enhancing autophagy through activation of AMPK signaling (increased Beclin1, LC3-II, and Bcl-2 expression and decreased Bax expression) and maintain cell viability [171]. Salvianolic acid B can promote the proliferation of 3T3-L1 cells and human-adipose-derived stem cells and enhance the differentiation of adipose-derived stem cells by increasing the expression of adipogenesis-related genes. In addition, it can improve the survival rate of nude mice with fat transplantation [172]. Salvianolic acid B improves testicular spermatogenesis by reducing ROS production by downregulating the expression of xanthine oxidase in rat models of testicular torsion/detorsion-induced ischemia/reperfusion injury [173]. In rat models of crush syndrome induced via bilateral hindlimb compression using a rubber tourniquet, treatment with salvianolic acid B led to a substantial improvement in survival by alleviating kidney and cardiac dysfunction, inflammation, and endothelial dysfunction by improving mitochondrial function and exerting antibacterial effects via neutrophil extracellular traps [174]. Salvianolic acid B exerts protective effects on cystine-stimulated HK-2 cells by upregulating antioxidant activity and the expression of antioxidant-related proteins, thus reducing the risk of cystine crystal formation in Slc7a9-knockout mice and protecting against cystine stone-induced injury [175]. In addition, it activates Akt signaling and the expression of the antiapoptotic protein Bcl-2, downregulates apoptosis in enterovirus 71-infected HeLa cells, and inhibits enterovirus 71 replication, demonstrating its therapeutic effects against hand, foot, and mouth disease [176]. In particulate matter (PM2.5)-stimulated mice and 16HBE human bronchial epithelial cells, salvianolic acid B can inhibit airway inflammation and oxidative stress by inhibiting the TLR4/MyD88/TRAF-6/NLRP3 pathway and its downstream molecules ERK1/2 and P38. In addition, the administration of salvianolic acid B via inhalation directly targets the drug to the target organs, thus improving its bioavailability, enhancing its efficacy, and reducing adverse systemic effects [177].

In mouse models of high-fat-diet-induced obesity, salvianolic acid B decreases body weight gain, increases insulin sensitivity, alleviates the serum levels of LPS and TNF-α, improves intestinal epithelial integrity, and increases the abundance of Gram-negative *Proteobacteria* and *Deferribacteres* by regulating the gut microbiota composition and the LPS/TLR4 signaling pathway [178]. In a study, the Illumina Hiseq 4000 platform was used to analyze mRNA, lncRNA, and circular RNA expression in mice with high-fat-diet-induced obesity. The results revealed that protein-coding genes upregulated by salvianolic acid B in white adipose tissue were involved in insulin-resistance-related pathways, whereas the downregulated genes mainly participated in the IL-17 signaling pathway. In addition, salvianolic acid B alleviated obesity by regulating anti-inflammatory-related factors and signaling pathways [179,180].

In a study, female CBA/J mice were allowed to mate with male DBA/2J mice to establish a spontaneous abortion model. The abortion rate and the expression of Nkp46 and the abundance of cytotoxic CD8+ T cells in the placenta of female mice significantly decreased after salvianolic acid B treatment, which was beneficial for immune modulation at the maternal–fetal interface during spontaneous abortion [181]. Supplementation of salvianolic acid B to mice exposed to γ-irradiation suppressed MDA, ROS, and Bax levels; increased the abundance of peripheral white blood cells, red blood cells, and platelets; improved thymus and spleen indices; and activated the Nrf2-mediated antioxidant pathway, which played a beneficial role in enhancing the hematopoietic and immune systems [182].

Salvianolic acid B prompts the proliferation, migration, and production of collagen type III in human gingival fibroblasts, which are the most important processes in wound healing [183]. In addition, it has demonstrated anticonvulsant and antiapoptotic effects in a pentylenetetrazole-induced seizure model through activation of the protein kinase B/cAMP response element-binding protein/brain-derived neurotrophic factor (AktCREB/BDNF) signaling pathway [184].

**Table 11:** Other effects.

Pharmacology	Experimental Subject	Experimental Dose	Animal Experimental Drug Delivery Method	Effects	Ref.
Protect the retina	High-glucose-induced primary Müller cells in the rat retina	40 μMol/L	None	Decrease: Bax Increase: Beclin1, LC3-II, p-AMPK, Bcl-2	[171]
Improve the fat transplantation survival	Human-adipose-derived stem cells 3T3-L1 Nude mouse fat transplantation	10, 50, 100 μMol/L 10, 50 μMol/L	None	Increase: Cell proliferation, Adipogenic differentiation	[172]
Protect the testicles	Rat testicular ischemia/reperfusion	10 mg/kg	Intravenous injection	Decrease: MDA Increase: Testicular weight, Seminiferous tubular diameter, Germ cell layer number	[173]
Improve crush syndrome	Comprised anesthetized rats with bilateral hindlimb compression by a rubber tourniquet	10, 20, 50 mg/kg	Intravenous injection	Decrease: IL-6, Cyt c, IL-1β, HMGB1, TNF-α, Nox Increase: SOD	[174]
Prevent cystine stones	Cystine-induced HK-2 cell Slc7a9 knockout mice	500 ug/L 60 mg/kg	Oral administration	Decrease: MDA, Cleavage of the eukaryotic eIF4G1 protein, Enterovirus 71 capsid protein viral polyproteins 1 Increase: SOD, GPx, Heat shock protein 90, Catalase	[175]
Treat hand, foot, and mouth disease	Enterovirus 71-induced HeLa cells	100, 10 μg/mL	None	Increase: Bcl-2, Cyclin-D1	[176]
Protect the airway	PM2.5-induced mice PM2.5-induced human bronchial epithelial 16HBE cells	2.5, 7.5, 15 mg/mL 2.5, 5, 10 μM	Nebulized inhalation	Decrease: MyD88, IL-1β, TNF-α, KC, TGF-β1, TLR4, TRAF-6, NLRP3, ROS, Phosphorylation-ERK1/2, Phosphorylation-p38 Increase: SOD, CAT, GSH, GSH-Px	[177]
Inhibit obesity	High-fat-diet-induced mice	100 mg/kg	Gavage administration	Decrease: Sterol regulatory element-binding protein 1c, Fatty acid synthase 1, Diacylglycerol O-acyltransferase 2, Insulin-induced gene 1 protein, Insulin-induced gene 2 protein, 3-hydroxy-3 methylglutaryl coenzyme A reductase, TG, TC, LDL, ALT, AST, Gram-negative *Desulfovibrionacea*, *Helicobacteraceae*, *Deferribacteraceae*, *Mucispirillum, Odoribacter*, TLR4, MYD88 Increase: HDL, *Lactobacillaceae*, *Adlercreutzia*, *Bifidobacterium*	[178]
Enhance immune system	CBA/J females mated with DBA/2J males as a spontaneous abortion mouse model	100 mg/kg	No mention	Decrease: TLR-2, TLR-4, NF-κB, IFN-γ, TNF-α, Nkp46, CD8+T Increase: Placental labyrinth area, SOD, Nrf2	[181]
	Mice received γ-irradiation	5, 12.5, 20 mg/kg	Intraperitoneal injection	Decrease: MDA, Bach1, ROS, Bax Increase: Peripheral white blood cells, Red blood cells, Platelets	[182]
Accelerate wound healing	Human gingival fibroblasts	25, 50, 75, 100, 150 µg/mL	None	Increase: Cell viability, Wound healing, Collagen III	[183]
Anticonvulsant	Pentylenetetrazole-induced rats	20 mg/kg	Oral administration	Decrease: Bax, cleaved caspase-3 Increase: Bcl-2	[184]

## 3. Safety

Salvianolic acid B at a concentration of ≤300 mg/kg is not toxic to pregnant rats, and salvianolic acid B at a concentration of 100 mg/kg is not toxic to embryo–fetus development. In addition, the administration of salvianolic acid B at the abovementioned concentrations does not induce significant genotoxicity [185]. Salvianolic acid B does not have hemolytic and coagulant effects on erythrocytes. Intravenous and intramuscular administration of salvianolic acid B does not cause significant vascular or muscular irritation in rabbits; however, toxicity is observed after it is administered via the tail vein at a high dose (750 mg/kg) (LD50 = 636.89 mg/kg; 95% confidence interval, 617.23–657.18 mg/kg), suggesting that salvianolic acid B is safe and can be used in clinical settings at appropriate concentrations [186]. A phase 1 clinical study (CTR20192236, CXZL0600063) has been completed in China for an injectable formulation of salvianolic acid B developed by Nanjing Hongqiao Pharmaceutical Technology Research Institute Co., Ltd (Nanjing, China). In a randomized, double-blind, placebo-controlled, single-center study, 47 healthy volunteers were randomly divided into 25, 75, 150, 200, 250, and 300 mg groups for single ascending dose identification. A total of 41 adverse events were observed in 24 (51.1%, 24/47) patients. For multiple ascending dose identification, 16 healthy volunteers were randomly divided into 150 and 300 mg groups, and 13 adverse events were observed in 8 (50.0%, 8/16) patients. Adverse events related to the treatment included increased levels of ALT (4.0%), bilirubin (2.0%), CK-MB (2.0%), brain natriuretic peptide (8.0%), and urine N-acetyl-β-D-glucosidase (4.0%), as well as dizziness (2.0%) and chest discomfort (2.0%). All adverse events were minor. Administration of salvianolic acid B up to a concentration of 300 mg in a single dose and 250 mg for 5 consecutive days showed excellent safety and tolerability in healthy Chinese volunteers [187].

Salvianolate, an injectable formulation, is used clinically for the treatment of stable angina pectoris in coronary heart disease. Magnesium lithospermate B is the magnesium salt form of salvianolic acid B, which is 80% of the ingredients [188]. In a 30-day-long toxicity analysis in Beagle dogs (Z20050248), hepatocytes and renal tubular epithelial cells in the high-dose group (320 mg/kg) were found to contain bilirubin and brownish-yellow drug-like substances, whereas lung and spleen tissues, mesenteric lymph nodes, the medullary sinus, and the intestinal mucosa were found to have phagocytosed drug-like macrophages. These organs and tissues were brown, and bone marrow hematopoietic tissues were mildly hyperplastic with some thrombosis and occasionally seen yellow drug-like material. Endothelial cells at the injection site were damaged, a fresh thrombus was formed in the blood vessels, and some thrombi were mechanized. *Salvia* polyphenolic acid salt as an injectable formulation has not been associated with a large number of rare and serious adverse events in clinical settings and has demonstrated a high safety profile [189,190]. But it is worth noting that because of salvianolate’s blood activation and other effects, physicians should be cautious of administering this drug to patients with bleeding tendencies [191].

## 4. Combination Therapy

In rat models of myocardial infarction induced via ligation below the left descending coronary artery, salvianolic acid B combined with ginsenoside Rg1 improves cardiac function through the maximum rates of pressure development for contraction and relaxation, reduces α-SMA expression and MMP-9 activity to reduce myocardial infarct size, prevents or delays the progression of cardiac remodeling, and improves cardiac structure and function [192]. In a study, the evaluation of fecal DNA and 16S rDNA high-throughput sequencing revealed that salvianolic acid B combined with ginsenoside Rg1 modulated the gut microbial composition, reduced the overall diversity of gut microbiota in feces, decreased blood glucose and lipid levels, and improved glucose tolerance in mice with high-fat-diet-induced obesity [193].

Salvianolic acid B combined with sodium tanshinone IIA sulfonate has demonstrated good inhibitory effects on both an LPS-induced inflammation model of THP-1 macrophages and a TGF-β1-induced lung fibrosis model of MRC-5 cells. The combination therapy modulates LPS–TLR4/NF-κB signaling pathway-related factors, thereby exerting anti-inflammatory effects, and inhibits TGF-β1/Smad pathway-related factors, thereby exerting anti-fibrotic effects. Both components have synergistic effects against fibrosis to some extent [194]. In a bovine serum albumin-induced coronary artery injury mouse model with Kawasaki disease, tanshinone IIA and salvianolic acid B inhibited myocardial tissue changes and reduced myocardial necrosis in mice by decreasing TNF-α, IL-6, and IL-1β levels [195].

Salvianolic acid B combined with hydroxysafflor yellow A protects against oxygen–glucose deprivation/reperfusion-induced damage in H9c2 cardiomyocytes by exerting anti-inflammatory, antioxidant, and antiapoptotic effects, accompanied by a decrease in TNF-α, IL-6, IL-1, MDA, LDH, and creatine kinase levels and an increase in SOD levels [196].

Salvianolic acid B and its magnesium salt *Salvia miltiorrhiza* polyphenolate can inhibit the entry of SARS-CoV-2 into Vero-E6 cells in vitro by blocking SARS-CoV-2 spike protein-mediated virus–cell membrane fusion [197] (Table 12).

## 5. New Dosage Forms and Drug Delivery Routes

Given that salvianolic acid B is very hydrophilic and has poor stability in water, achieving long-term stable release and ideal therapeutic effects is difficult. Therefore, it is important to develop a delivery system with high drug-loading capacity and slow drug-releasing ability to improve the efficacy of salvianolic acid B (Table 13).

In a study, HPLC revealed that polyethyleneimine (PEI)-modified graphitic carbon nitride nanosheets achieved a maximum loading rate of 327.4% for salvianolic acid B and released the drug slowly with a 7-day cumulative release rate of 79.2%, with good biocompatibility and high stability. Salvianolic acid B at concentrations below 800 μg/mL had minimal effects on the mortality and hatching rate of zebrafish embryos and did not affect hatching and morphological changes [198]. In a recent study, carboxymethyl panax notoginseng polysaccharide–chitosan polyelectrolyte complex nanoparticles loaded with salvianolic acid B/doxorubicin were prepared using the polyelectrolyte titration method. HPLC revealed a significantly slow drug release effect of the nanoparticles. In 4T1 mouse breast cancer cells and H9c2 mouse cardiomyocytes, the nanoparticles could simultaneously kill tumor cells and protect cardiomyocytes [199]. In another study, salvianolic acid B was incorporated into nanoparticles using polybutylcyanoacrylate as a carrier, which was modified with ice chips. The in vitro drug release was measured via UV spectrophotometry, and the cumulative release at 12 h reached approximately 77%, which demonstrated a slow release effect. Analysis of tissue distribution in mice showed that the concentration of the nanoformulation in the brain of mice was significantly higher than that of the reference formulation. In addition, the peak concentration of the drug was 3.95 times higher in the nanoformulation group than in the reference formulation group, which increased the concentration of the drug in the brain and enhanced the brain-targeting ability of the drug [200]. Ye et al. synthesized amphiphilic drug−drug conjugates by covalently linking dexamethasone and salvianolic acid B through an ester or amide bond. The conjugates could self-assemble into nanoparticles with ultrahigh drug-loading capacity and favorable stability. In cisplatin-induced ototoxicity in zebrafish and guinea pig models, HPLC determined drug concentration in cochlear samples and indicated that the conjugates and nanoparticles could reach the cochlear tissue and almost completely restored hearing in experimental animals at all tested frequencies. The conjugates and nanoparticles exhibited synergetic and better anti-ototoxic effects in both in vivo and in vitro models without causing adverse effects and did not elicit inflammatory responses in the cochlear tissue, preliminarily demonstrating good biosafety [201]. Mice with 4-nitroquinoline-N-oxide-induced oral tongue carcinogenesis were treated with a phospholipid complex load of nanoparticles encapsulated with salvianolic acid B, and the sustained release of salvianolic acid B promoted more potent anti-proliferation and cell cycle arrest responses, demonstrating good efficacy [202].

Lin et al. fabricated composite scaffolds comprising poly (lactic-co-glycolic acid) and tricalcium phosphate (PLGA/β-TCP) using a low-temperature rapid prototyping technique and they were encapsulated with salvianolic acid B. HPLC revealed that the drug was steadily released from the scaffolds. The aliquot of released salvianolic acid B could enhance bony fusion through the promotion of osteogenesis and angiogenesis in EA-hy9.26 cells and rat models of spinal fusion [203]. Liu et al. developed poly (lactic acid) (PLA)/graphene oxide (GO)/salvianolic-acid-B-loaded porous biomimetic composite scaffolds via thermally induced phase separation. UV–visible spectrophotometry revealed that the biomimetic scaffolds controlled the slow release of salvianolic acid B and had cytocompatibility. The addition of a small amount of salvianolic acid B significantly promoted MC3T3-E1 cell proliferation, inducing osteogenic differentiation and ALP activity. Altogether, the scaffolds demonstrated good hemocompatibility and may be suitable for use in tissue engineering [204]. In another study, salvianolic acid B was combined with GO and added to a silk fibroin solution to form a scaffold structure using the freeze-drying method. X-ray photoelectron spectroscopy and Fourier-transform infrared spectroscopy (FTIR) revealed that the scaffolds could sustainably release salvianolic acid B. In addition, the scaffolds promoted the adhesion and osteogenic differentiation of rat BMSCs and enhanced cell migration and tubule formation in EA-hy9.26 cells. After 8 weeks of implantation of the silk fibroin/GO/salvianolic acid B scaffolds in rats with cranial defects, the defect area showed more new bones and stronger angiogenesis than in rats implanted with silk fibroin and silk fibroin/GO scaffolds. Altogether, the silk fibroin/GO/salvianolic acid B scaffolds contributed to the growth of new blood vessels and accelerated osseointegration after implantation into the defect site [205]. Ji et al. developed chitosan/hydroxyapatite bone scaffolds loaded with salvianolic acid B with controlled release and effective bioactivity. HPLC revealed that the release of salvianolic acid B from the scaffold was stable and continuous for 8 weeks in vitro. The osteogenic and angiogenic bioactivities of the salvianolic-acid-B-loaded chitosan/hydroxyapatite scaffolds were effective in rabbit models of radial defects in vivo and in MC3T3-E1 cell and HUVECs in vitro [206]. Liu et al. developed PLA/GO/salvianolic acid B/aspirin (ASA) dual-drug-loaded biomimetic composite scaffolds via thermally induced phase separation. X-ray diffraction and FTIR showed that the addition of salvianolic acid B increased the hydrophilicity of the scaffolds, whereas an increase in ASA reduced the porosity and contributed to the slow release of salvianolic acid B. The dual-drug-loaded scaffolds had good hemocompatibility and synergically promoted the proliferation of MC3T3-E1 cells and enhanced ALP activity [207].

A study demonstrated that polydopamine nanoparticles (PDA) loaded with salvianolic acid B and elastin-mimic peptide hydrogel (EMH) had excellent biocompatibility and low viscosity. After intramyocardial injection of these hydrogels into rat models of myocardial infarction induced via coronary artery ligation, the hydrogels remained in the ventricular wall for a long period. Salvianolic acid B could be gradually and slowly released, and rats with ischemic myocardial infarction recovered from heart failure after four weeks of treatment [208]. In another study, 1% hyaluronic acid methacryloyl (HAMA) hydrogels were used as carriers, and BMSCs and salvianolic acid B were co-encapsulated into the hydrogels. The percentage of cell survival within the hydrogels was significantly higher. The pinprick method was used to establish rat models of disc degeneration. Injection of the hydrogel mixture into the intervertebral disc delayed the progression of disc degeneration [209]. Recently, Cao et al. developed methacrylate-based gelatin hydrogels loaded with salvianolic acid B. The establishment of intervertebral disc degeneration model rats by acupuncture, after injections of salvianolic-acid-B-loaded hydrogels for four weeks, inhibited oxidative stress and inflammation in the degenerated disc tissue, and effectively alleviated disc degeneration [210]. Luo et al. and Xu used the thin film dispersion–pH gradient method to prepare liposomes co-encapsulating tanshinone IIA and salvianolic acid B, which were further loaded with oxidized hyaluronic acid/succinyl chitosan to produce hydrogels with good sustained release properties (HPLC analysis). The good transdermal absorption and dermal retention properties of these hydrogels were validated in rat skin, which helped reduce the frequency of drug administration. The hydrogels were non-irritating to the skin of New Zealand rabbits after single or multiple administrations and had good safety during transdermal administration [211,212].

Studies have used TAT (a membrane-penetrating peptide)-modified liposomes to encapsulate salvianolic acid B via pH gradient inverse evaporation. The cumulative in vitro 24-hour release rate is 62.49%, as measured via HPLC, with no abrupt release effect and an evident slow release effect, which enhances the skin penetration and dermal targeting of the drug. The transdermal performance of the liposomes has been validated in isolated rat skin in vitro, and the liposomes have been found to be non-irritating and non-sensitizing to human skin fibroblasts (HSFs) [213,214,215]. Liu et al. used the thin film dispersion–pH gradient method for preparing liposomes co-encapsulating tanshinone IIA and salvianolic acid B. The liposome had good stability, sustained drug release behavior, and good transdermal properties and exerted inhibitive effects on the proliferative, migratory, and invasive capabilities of HSF cells [216]. Mice were injected with breast cancer 4T1 cells and embryonic fibroblast NIH3T3 cells to establish a mouse breast cancer model. PEGylated salvianolic acid B liposomes prepared by ethanol injection enhanced the permeation distribution and cytotoxicity of nanoparticles in three-dimensional multicellular tumor spheroids, prolonged salvianolic acid B circulation, had a slow release effect, improved tumor immunity and the fibrotic microenvironment, and caused no significant toxicity to other tissues [217].

Zhang et al. prepared a lipid emulsion of salvianolic acid B via high-speed dispersion combined with ultrasonic emulsification, which delayed the release of salvianolic acid B and improved its oral bioavailability. The structure of hepatocytes in mice with acetaminophen-induced acute liver injury was normalized after the lipid emulsion was administered via gavage [218].

In a study, L-leucine was used as an excipient to prepare a salvianolic-acid-B-containing dry powder inhaler using the spray-drying method, targeted to the lungs in idiopathic pulmonary fibrosis rats induced by bleomycin saline solution through tracheal non-invasive lung drug delivery. The inhaler did not cause irritation to the lungs and overcame the disadvantages of poor oral absorption and low bioavailability of salvianolic acid B. After 1 h of pulmonary administration, the concentration of salvianolic acid B in lung tissues was 2940.62 ± 117.04 ng/g, indicating that the inhaler has certain advantages in the treatment of pulmonary diseases [219]. Guo et al. developed a salvianolic-acid-B-containing microemulsion that enhanced the skin-penetrating ability of salvianolic acid B, had high molecular weight, and was hydrophilic. The microemulsion improved barrier function, alleviated disease severity, reduced acanthosis, inhibited epidermal proliferation, and increased skin hydration in mice with topical imiquimod-induced psoriasis [220].

Recent studies have shown that microemulsion gel + microneedle administration has good transdermal properties and a slow release effect, and its administration to treat mice toe swelling has anti-inflammatory and sebaceous gland secretion control effects [221]. After intranasal administration of salvianolic acid B in rats, compared with intravenous injection, the Cmax levels in the blood and brain were significantly prolonged and increased by nearly 2.03 and 1.86 times, respectively, showing that salvianolic acid B has a certain brain-targeting mechanism, which could become a new drug system for the treatment of brain diseases [222].

## 6. Conclusions

According to the China Cardiovascular Health and Disease Report (2022), the prevalence of cardiovascular disease (CVD) in China is on the rise. The projected number of current CVD patients is 330 million, of which 13 million are strokes, 11.39 million are coronary heart disease, 8.9 million are heart failure, 5 million are pulmonary heart disease, 4.87 million are atrial fibrillation, 2.5 million are rheumatic heart disease, 2 million are congenital heart disease, 45.3 million are peripheral arterial disease, and 245 million are high blood pressure. CVD is the leading cause of death among urban and rural residents, and in 2020, CVD accounted for 48.00% and 45.86% of causes of death in rural and urban areas, respectively. Two out of every five deaths are due to CVD. Currently, salvianolic-acid-B-based injections of Salvia miltiorrhiza polyphenolic acid salt for injection and salvianolic acids for injection are both included in the National Basic Medical Insurance, Work Injury Insurance, and Maternity Insurance Drug Catalogs and the Chinese Guidelines for the Combined Diagnosis and Treatment of Cerebral Infarction with Chinese and Western Medicine 2017. Salvianolic acids for injection are used for patients recovering from cerebral infarction in secondary and above medical institutions, with sales of RMB 91.89 million in 2022 [223]. Salvia miltiorrhiza polyphenolic acid salt for injection is used for patients in secondary and above medical institutions who have a clear diagnosis of stable angina of coronary heart disease, with sales of RMB 443.3 million in 2022 (pdb.pharmadl.com). It can be seen that the preparation based on salvianolic acid B has an excellent therapeutic effect on CVD and has good market potential. A study on the safety, tolerability, and pharmacokinetics of salvianolic acid B for injection lasted for 14 months, which was highly recognized by the sponsor and CRO, indicating that the level of phase I clinical research of salvianolic acid B for injection was internationally recognized and had good potential [187].

*Salvia miltiorrhiza* polyphenolic acid salt for injection (Z20050248) (containing 80% salvianolic acid B) is clinically used for treating stable angina pectoris in coronary heart disease in China. In addition, salvianolic acid B for injection (CTR 20192236, CXZL 0600063), which is currently applied for marketing, is indicated for coronary heart disease and angina pectoris in the first phase of clinical trials. Salvianolic acid B exerts excellent protective effects not only on damaged organs and tissues, but also on different types of tumors, inflammation, depression, and spinal cord injury by improving the corresponding indices. Studies have demonstrated that salvianolic acid B can protect against coronavirus disease 2019 [224,225], exerts anti-aging effects [226,227], and can be used in the treatment of uremic syndrome [228]. Recently, commercially available dosage forms based on salvianolic acid B mainly include injections containing *Salvia miltiorrhiza* polyphenol acid for injection and salvianolic acids for injection. Additionally, there are capsules such as Shenkangning capsule [229], Yigan Fuzheng capsule [230], ChangMaiLe I capsule [231], Xinkeshu capsule [232], Naoxintong capsule [233]; Granules: Lutai Danshen Baishao granules [234], Danshen formula granules [235], and Dangua Humai granules [236]; mixtures such as Heying Mixture [237]; tablets such as compound realgar and natural indigo tablets [238], SheXiang XinNaoTong tablets [239], Fufang xiatianwu tablets [240], and Jianyao Migu tablets [241]; pills such as compound Danshan dripping pills [242], Jingshen Pills [243], Zhongfenghuichun pilule [244], and Qingbutongluo Pills [245]; oral liquids such as Ruanmailing Oral Liquid [246] and Huolisu Oral Liquid [247]; suppositories such as Shenkang Suppositories [248]; and ointments such as Kun-an cream [249]. It has been reported that the content of salvianolic acid B in the above dosage forms was determined by HPLC as a quality index.

Salvianolic acid B can be administered via various administration routes. Of the 77 in vivo studies analyzed in this review, 41 studies used intraperitoneal injection, 20 studies used gavage administration, 8 studies used oral administration, 2 studies used nebulized inhalation, 1 study used injection passed through the annulus fibrosus, and 1 study used a local injection route. Salvianolic acid B has a wide range of pharmacological effects and great excavation value. Each route of drug delivery has its advantages and scope of application for different diseases. Comparisons of the efficacy of salvianolic acid B administered via different routes are rarely reported. At present, the primary routes of administration of salvianolic acid B in animal experiments are intraperitoneal injection and gavage; therefore, further studies are warranted to identify more effective routes of administration. Future experimental designs can use different routes of administration, all acting on the same type of disease, to analyze differences in the effects of different routes of administration from the perspective of bioequivalence, which may provide a reference for further studies on the mechanism of action of salvianolic acid B.

In clinical settings, the efficacy of a single drug may be limited, and the combination of two or more drugs is usually used to exploit the synergistic effects of drugs, which improves the efficacy while reducing or delaying drug resistance and alleviating toxic effects. The combination of salvianolic acid B with ginsenoside Rg1, tanshinone IIA, and hydroxysafflor yellow A can effectively treat heart-related diseases. Salvianolic acid B combined with ginsenoside Rg1 can modulate the gut microbiota composition. Combined with sodium tanshinone IIA sulfonate, it exerts synergistic anti-fibrotic effects. Combined with its magnesium salt Salvia miltiorrhiza polyphenolate injection, it exerts anti-viral effects. Given the complexity and diversity of diseases, a combination of two active ingredients is more effective.

Salvianolic acid B has rich pharmaceutical value. It is safe and effective, with few side effects. Salvianolate injection has been widely used in clinical practice. However, owing to its high relative molecular mass and poor lipid solubility, the absolute oral bioavailability of salvianolic acid B is low, which greatly limits its therapeutic potential. Many studies have attempted to develop new dosage forms and routes of administration of salvianolic acid B. Composite scaffolds, nanoparticles, liposomes, and hydrogels are used as carriers for the controlled release of salvianolic acid B. Two new dosage forms, dry powder and microemulsion, combined with new drug delivery methods have also shown significant therapeutic effects. These new dosage forms improve the stability and bioavailability of salvianolic acid B and allow for direct drug action at the target site, independent of other factors in the body, while avoiding hepatic first-pass effects, hepatic and renal toxicity, and gastrointestinal reactions. New routes of administration include intracorporeal implantation, intramyocardial injection, injection into the intervertebral disc, noninvasive tracheal administration, and the direct delivery of salvianolic acid B to the focal site, which is a promising application route for the formation of a high-level drug reservoir with long-lasting efficacy.

However, there are still gaps in the clinical research on the combination therapy, new dosage forms, and new routes of administration of salvianolic acid B. How to translate theoretical research into practical clinical applications is a challenge to solve. The research on pharmacological action mechanisms of animal or cell experiments mostly focuses on a single target or signal pathway. In the future, genomics, proteomics, metabolomics, and other new technologies should be applied to the in-depth study of salvianolic acid B combination therapy, new dosage forms, new routes of administration, its interactions, and its pharmacological mechanisms of action. Only by clarifying its specific mechanism of action can we provide a basis for the accurate positioning of subsequent clinical research.

Only a few studies have used the combination of salvianolic acid B and other active ingredients of traditional Chinese medicine to develop new dosage forms or delivery routes of salvianolic acid B. Therefore, combining new drug delivery systems with traditional Chinese medicine is a promising approach to developing drug delivery systems with good performance to improve the therapeutic effects and promote the more rational development and use of salvianolic acid B.

## Figures and Tables

**Table 1:** Antitumor effects of salvianolic acid B.

Pharmacology	Experimental Subject	Experimental Dose	Animal Experimental Drug Delivery Method	Effects	Ref.
Antitumor effects	Colon cancer cells RKO Prostate cancer cells PC3 Mice inoculated with MC38 colon cancer cells	5, 10, 20 μMol/L 10, 20 mg/kg	Intraperitoneal injection	Decrease: PD-L1, USP2 Increase: CD8+ T	[34]
	Human-derived HCT116 colon cancer cell line	50, 100, 200 μMol/L	None	Decrease: LC3-I, p62, AKT, mTOR Increase: LC3-II, Atg5	[35]
	TGF-β1-induced NSCLC A549 cells	25, 50, 100 µM	None	Decrease: N-cadherin, vimentin, Snail, cyclin B1, LC3α, p62, Bcl-2, ERK1/2, JNK1/2, p38, p-Smad2, p-Smad3 Increase: p21, LC3β, Beclin1, Bax, caspase-3, cleaved caspase-3	[37]
	NSCLC NCI-H2030 and NCI-H1650	100, 200, 300, 400, 500, 600 μM	None	Decrease: Cell migration, Cell invasion, Glucose uptake, Lactate production, Enolase activity, ATP, Oxygen consumption rate, Extracellular acidification rate, Pyruvate kinase M2, LDHA, GLUT1, β-catenin, E-cadherin	[38]
	Normal gastric mucosa epithelial cell line GSE1 Gastric cancer cells AGS and AGS/cisplatin	25, 50, 100, 200, 250 µM	None	Decrease: Cell viability, Cell clone, Cell migration, Cell invasion, Vimentin, N-cadherin, MMP2, MMP9, EMT formation Increase: ROS, E-cadherin	[39]
	Human hepatocellular carcinoma cell lines HepG2 and HCCLM3	100, 200, 300, 400, 500, 600 μM	None	Decrease: N-cadherin, vimentin, Mortalin, MMP2, MMP9, p-STAT3Y705, Ac-STAT3K685 Increase: RECK, E-cadherin	[40]
	3-Aminopropionitrile induced in rats	20 mg/kg	Intraperitoneal injection	Decrease: MMP -2, MMP -9, CD8+, ROS, MDA, IL-1β, IL-6, IL-8, TNF-α, JAK2, STAT3, p-JAK2, p-STAT3 Increase: CD4+, CD4+/CD8+, IgA, IgG, IgM, SOD	[41]
	Triple-negative breast cancer cells MDA-MB-231 and MDA-MB-468	3.12, 6.25, 12.5, 25, 50 μM	None	Decrease: Cell migration, Cell invasion, E-cadherin, Cytokeratin 19 Increase: N-cadherin, Vimentin, p-Snail/Snail, p-Slug/Slug	[42]
	Human MDA-MB-231 breast cancer cells	0–100 μM	None	Decrease: MMP-9	[43]
	Mice inoculated with 4T1 murine-derived breast cancer cells and MDA-MB-231 human-derived breast cancer cells	100, 40 mg/kg	Intraperitoneal injection	Increase: NG2+, αSMA+, Collagen IV+, VE-Cadherin+, Claudin 5+	[44]
	Human breast cancer adenocarcinoma MCF-7 Mice injected with Ehrlich solid carcinoma cell line	0 to 1.0 mg/mL 25 mg/kg	Intraperitoneal injection	Decrease: TNF-α, MMP-8, Cyclin D1, VEGF, COX-2, MDA Increase: caspase-3, P53, GSH	[45]
	Endometrial cancer MFE-280 cells	0.5, 1, 2.5 μMol/L	None	Decrease: Cell clone formation rate, p-PI3K/PI3K, p-AKT/AKT Increase: Apoptosis rate, Beclin1, LC3-II/LC3-I, ATG7, Mitochondrial membrane potential	[46]
	Ovarian cancer cells ID8	0.87, 1.74, 3.48, 6.96, 13.92 μM	None	Decrease: Cell proliferation, Cell cloning ability, PI3K110β, PDPK1, p-PDPK1 (Ser241), pan-Akt, p-Akt1 (Ser473), p-GSK3β (Y216)	[47]
	Ovarian cancer cells ID8 Mice inoculated with ovarian cancer cells ID8	3.75, 0.9375, 1.875, 3.75, 7.5, 15 μM	None	Decrease: Cell proliferation, Cell cloning ability, p-IκBα, IκBα, p-p65, p65	[48]

**Table 2:** Effects of salvianolic acid B on the lungs.

Pharmacology	Experimental Subject	Experimental Dose	Animal Experimental Drug Delivery Method	Effects	Ref.
Effects on the lung	TGF-β1-induced human lung epithelial A549 cells	10, 20, 40, 80, 160 μMol/L	None	Decrease: Cell proliferation, FN, Collagen type I Increase: E-calcium viscosity	[50]
	LPS-induced human acute monocytic leukemia cell line THP-1 TGF-β1-induced human lung fibroblast cell line MRC-5	20, 50, 100, 200, 300, 400, 500, 600, 700, 800, 900 μg/mL	None	Decrease: TNF-α, IL-1β, IL-6, α-SMA, Collagen 1α1	[51]
	Bleomycin-induced rats	1, 10, 25, 40, 50 mg/mL	Nebulized inhalation	Decrease: Hydroxyproline, Collagen-1, Tissue factor/Coagulation factor VII, Activated coagulation factor X, Thrombin–antithrombin complex, Fibrinogen degradation product, Plasminogen activator inhibitor-1, PAR-1, p-PKC Increase: Coagulation factor Ⅱ, Coagulation factor X, Tissue-type plasminogen activator, Urokinase-type plasminogen activator	[52]
	Nasal drops of Staphylococcus aureus in young rats	5, 10, 20 mg/kg	Intraperitoneal injection	Decrease: IL-1β, IL-6, TNF-α, NF-κBp65, MDA Increase: Arterial partial pressure of oxygen, Oxygenation index, SOD	[53]

**Table 4:** Effects of salvianolic acid B on the heart.

Pharmacology	Experimental Subject	Experimental Dose	Animal Experimental Drug Delivery Method	Effects	Ref.
Effects on the heart	Mice	30, 60, 120 mg/kg	Gavage administration	Decrease: MDA, Bacterbidetes, Proteobacteria, Verrucomicrobia, Actinobacteria Increase: GSH, SOD, Firmicutes A, Gordonibacter	[68]
	Mice were subjected to cardiac arrest induced by an intravenous injection of potassium chloride, followed by cardiopulmonary resuscitation H9c2 cardiomyocytes with hypoxia/reoxygenation	20 mg/kg 10 μM	Intravenously	Decrease: Bax, caspase-3, SOD, NADPH oxidase activity, Keap1 Increase: Bcl-2, Bcl-2/Bax, HO-1, NQO1	[69]
	H_2_O_2_-induced cardiomyocyte H9c2 cells Ligation of the left anterior descending coronary artery in rats	5, 10, 20 μMol/L 8, 32 mg/kg	Intravenously	Decrease: LDH, CK-MB, cTnI, ROS, IL-1β, NLRP3, Caspase-1, ASC, p-AMPKα/AMPKα, PGC-1α Increase: Mitochondrial membrane potential	[70,71,72,73]
	Hypoxia-induced rat cardiomyocytes H9C2	1, 5, 10, 15, 20, 25, 30, 35 μMol/L	None	Decrease: LDH, cTnI, IL-1β, NLRP3, TLR4, Myd88, IRAK1, NF-κB	[74,75,76]
	H9c2 hypoxia/reoxygenation injury in cardiac myocytes	50 μMol/L	None	Decrease: LDH, ROS, LC3-II/LC3-I, cleaved caspase-3, NIX Increase: ATP, Mitochondrial membrane potential	[77]
	H9c2 cells with oxygen and glucose deprivation	1, 5, 10 μM	None	Decrease: Bax, caspase-3, caspase-9, MEG3, P53 Increase: cyclin D1, AMPK	[78]
	Ischemia/reperfusion of the left anterior descending branch of the ligated coronary artery in rats Cardiomyocyte H9c2 oxygen–glucose deprivation/reperfusion	200, 100, 50 μM 30, 60 mg/kg	Intraperitoneal injection	Decrease: LDH, CK-MB, C-HMGB1, ac-HMGB1, TLR4, NF-κB, Bax Increase: SIRT1, Bcl-2, N-HMGB1	[79]
	Ischemia/reperfusion of coronary artery ligation in rats	15, 60 mg/kg	Intraperitoneal injection	Decrease: L-LDH, CK-MB, TNF-α, IL-18, IL-β, TLR4 Increase: PI3K, AKT	[80,81]
	Ligation of the left coronary artery in rats	40 mg/kg	Intraperitoneal injection	Decrease: CK-MB, cTnI, LDH, MDA, ROS, Iron ion content, Area of Perl blue positivity Increase: GSH, Cx43	[82]
	LPS-induced bone-marrow-derived macrophages Myocardial ischemia/reperfusion in mice induced by ligation of the left anterior descending artery	80 mg/kg 5, 10, 20 μM	Gavage administration	(M1 biomarkers) Decrease: IL-6, iNOS, CCL2, TNF-α (M2 biomarkers) Decrease: RagD, mTORC1 Increase: Arg1, Clec10a, Mrc, CX3CR1, CD36, MerTK, IL-10	[83]
	Rats undergoing cecum ligation and perforation	6, 12, 24 mg/kg	Intravenously	Decrease: RagD, mTORC1, Myocardial necrosis markers troponin T, CK-MB, cTnI, IL-1β, IL-6, TNF-α, MDA Increase: SOD, LC3II/LC3I, Beclin-1	[84]
	AC16 cardiomyocytes were treated with ischemia/reperfusion The rat coronary artery was clamped with a plastic tube, and then the coronary artery was restored by releasing the clamp.	10, 25, 50 μM 20, 40, 60 mg/kg	Intravenously	Decrease: Apoptotic, ROS, MDA, TRIM8 Increase: SOD	[85]
	Streptozotocin injection in rats	12.5, 25, 50 μMol/L	Gavage administration	Decrease: α-SMA, Collagen I, Collagen III, Bcl-2 Increase: RhoA, ROCK1, Bax, Beclin1, LC3-I, LC3-II	[86]
	Streptozotocin-induced mice HUVECs under hypoxia	15, 30 mg/kg 50–100 μg/mL	Intraperitoneal injection	Decrease: ECM, Collagen I, Collagen III, IGFBP3, AKT phosphorylation, ERK phosphorylation Increase: VEGF, VEGFR2	[87]
	High-glucose-induced cardiac fibroblasts in rats	12.5, 25, 50 μMol/L	None	Decrease: Collagen fiber deposition, α-SMA, β-catenin, p-GSK 3β	[88,89]
	High-sugar, high-fat diet combined with streptozotocin injections induced in rats	160, 80 mg/kg	Gavage administration	Decrease: Fasting Glucose, CK-MB, Bax, caspase-3 Increase: Bcl-2	[90]
	Hydrogen peroxide intervention in rat bone marrow mesenchymal stem cells	50, 100, 150, 200 μg/L	None	Decrease: CK, LDH, MDA Increase: SOD Decrease: Keap1 Increase: Nrf2, Bcl-2/Bax, GATA4 genes, cTnT	[92]
	The left common carotid artery was dissected and ligated near the carotid bifurcation using suture to induce intima formation in mice Ang II-induced mouse aortic vascular smooth muscle cells	7 mg/kg 2.5, 5, 10 μMol/L	Intraperitoneal injection	Decrease: KLF5, cyclin D1, miR-146a	[93]
	Tunicamycin-induced bone-marrow-derived endothelial progenitor cells	10, 20 μM	None	Decrease: ROS, TXNIP, NLRP3, Cleavage caspase-1, IL-1β, IL-18, Caspase, Syndencan-4 Increase: HO-1, SOD2, p-AMPKα, KLF2	[94]

**Table 5:** Effects of salvianolic acid B on blood vessels.

Pharmacology	Experimental Subject	Experimental Dose	Animal Experimental Drug Delivery Method	Effects	Ref.
Effects on blood vessels	PTK787 causes vascular damage in zebrafish	10, 20, 40, 80 μg/mL	None	Increase: Generation of blood vessels	[95]
	Mice with hind limb ischemia model Mouse bone-marrow-derived macrophages isolated from the femur and the tibia bone marrow cells treated with salvianolic acid B, then collected the culture supernatant to culture HUVECs	15 mg/kg 1, 5, 25 μM	Intraperitoneal injection	Increase: M2 Polarization, Cell migration, Tube formation	[96]
	High-glucose-induced HUVECs exposed to carbonyl cyanide m-chlorophenyl hydrazone C57BL/KsJ db/db mice	5, 10, 30, 50, 80, 100 μM 50 mg/kg	Oral administration	Decrease: Bax, Beclin1, Parkin, PTEN Induced Kinase 1 (Pink1), TC, TG, LDL-C, HDL-C, Fasting blood glucose, Fasting insulin, Homeostasis model assessment insulin resistance Increase: Bcl-2, Mitochondrial activity, Ca^2+^	[97]
	Ang II-induced HUVEC	25, 50, 100, 200, 400, 800 μM	None	Decrease: ROS, MDA, NOX2 Increase: NO, Intracellular calcium ions, ERK1/2, p38, JNK, PI3K, AKT, NRF2, HO-1	[98]
	High-fat diet fed to LDLR knockout mice ox-LDL-induced macrophage RAW264.7 in mice	1.25, 2.50, 5.00 mg/L 25 mg/kg	Intraperitoneal injection	Decrease: TC, TG, LDL-c Increase: AXL, MERTK, TYRO3, MFGE8	[102]
	High-fat diet fed to LDLR knockout mice LPS-induced macrophage RAW264.7 in mice	200 μL 1.25, 2.5, 5 μg/mL	Intraperitoneal injection	Decrease: IL-6, IL-1β, TNF-α, TC, TG, LDL-c, STAT3, IκB, NF-κB	[103]
	High-fat diet fed to LDLR knockout mice ox-LDL-induced or LPS-induced RAW264.7 cells	25 mg/kg 1.25, 2.5, 5 μg/mL	Intraperitoneal injection	Decrease: TC, TG, LDL-C, AST, ALT, IL-6, IL-1β, TNF-α, VCAM, iNOS, p-JNK, p-P38, p-ERK 1/2, p-IκB, p-NF-κB p65	[104]
	ApoE-/- mice with high-fat-diet ox-LDL-induced Ecs and pericytes	30, 25 μg/mL 30 mg/kg	Intraperitoneal injection	Decrease: YAP, TAZ, JNK, Nuclear NF-κB P65, Total P65, TNF-α, ROS, MDA, TG, TC, LDL-C, IL-6, IL-1 β, TNF-α Increase: p-YAP, p-TAZ, SOD, Gpx	[105]
	RAW264.7 macrophages	50, 100, 150, 200, 250, 300, 350 μM	None	Decrease: iNOS, TNF-α, IL-6, p62, NF-κB p65 Increase: Arg-1, IL-10, LC3-II, Beclin-1, p-AKT, p-mTOR	[106]
	Cholesterol crystals stimulate RAW264.7 macrophages	40, 80, 120, 160, 200, 240, 280, 320, 360 µM	None	Decrease: TNF-α, IL-6, cleaved-caspase 3/pro-caspase 3, Bax/Bcl-2, p62 Increase: LC3 II, Beclin-1	[107]
	H_2_O_2_-induced HUVECs	5, 10, 20 µg/ml	None	Decrease: p62, Phosphorylation of mTOR, caspase-3, cytochrome c Increase: LC3-II, Beclin-1, Phosphorylation of AMPK	[108]
	Ang II-induced rat thoracic aortic outer membrane	10^−5^ mol/L	None	Decrease: ROS, Cell migration capacity, α-SMA	[109]
	High-phosphorus-induced vascular smooth muscle cells in rats	3, 10, 30 nmol/g	None	Decrease: Runx2, OPN Increase: Beclin-1, LC3-I/II, Calponin, SM22	[110]
	TNF-α-induced human EA.hy 926 cells Abdominal aorta of the rats	1, 5, 25, 50 μM	None	Decrease: PAI-1, Factor IIa, Factor Xa, Tissue factor, p-NF-κB p65, p-p38 MAPK, pJNK Increase: Tissue plasminogen activator	[113]

**Table 6:** Effects of salvianolic acid B on the kidneys.

Pharmacology	Experimental Subject	Experimental Dose	Animal Experimental Drug Delivery Method	Effects	Ref.
Effects on the kidney	Iopromide-induced damage to HK-2 human renal cortical proximal tubular epithelial cells	10, 50, 100 μMol/L	None	Decrease: Apoptosis, Bax/Bcl-2, cleaved caspase-3, ROS, Caspase-1, NLRP3, TLR4, p-NF-κB, IL-18, IL-1β, TNF-α, ASC, GRP78, p-eIF2α, p-JNK, CHOP Increase: Cellular mitochondrial membrane potential, Cellular viability	[114,115,116]
	Iopromide-induced HK-2 human renal proximal tubular epithelial cells	1, 10, 50, 100, 200 μMol/L	None	Decrease: ROS, p-ERK1/2, Bax, cleaved Caspase-3 Increase: p-Akt, Klotho, Bcl-2	[117]
	Modified chronic serum disease method for induction of rats	100 mg/kg	Gavage administration	Decrease: Serum creatinine, Urea nitrogen, Glomerular cell apoptosis, Caspase-3, Bax, Bax/Bcl-2, IL-1β, IL-6, IL-18, TNF-α, NF-κB p65 Increase: Bcl-2	[118]
	Rats injected with cationized calf serum albumin and Freund’s incomplete adjuvant	50, 100 mg/kg	Gavage administration	Decrease: Serum creatinine, Urea nitrogen, Urine protein, MDA, C3 deposition, IgG deposition, IL-1β, IL-6, p62 Increase: GSH, SOD, LC3 II/LC3 I, Beclin1	[119]
	Unilateral ureteral obstruction in rats	100 mg/kg	Gavage administration	Decrease: Fe^2+^, MDA, Collagen-I, Collagen-III Increase: SOD, Nrf2, Gpx4	[121]
	High-glucose-induced rat proximal renal tubular epithelial cell line NRK-52E	1, 5, 10, 20, 50, 100 μMol/L	None	Decrease: α-SMA, p-Akt Increase: PPARγ, PTEN, E-cadherin	[122]
	Rats subjected to unilateral nephrectomy TGF-β1-induced HK-2 cells	200, 100, 50 mg/kg 100 μM	Oral administration	Decrease: Fibronectin, α-SMA, TGF-β, Beclin1, LC3B Increase: p62, Sirt1	[123]
	Unilateral nephrectomy in mice with contralateral renal artery clamping of the renal tip Hypoxia-reoxygenated renal tubular epithelial HK-2 cells	200, 100, 50 mg/kg 80, 40, 20 μM	Gavage administration	Decrease: IL-1β, TNF-α, Caspase-1, MDA, NLRP3, TXNIP, Keap1, GSDMD Increase: HO-1, SOD, GSH	[124]
	Cationic bovine serum albumin-induced rats LPS-induced human mesangial cells	100 mg/kg	Oral administration	Decrease: TNF-α, IL-6, IL-1β, IL-2, CD68 Increase: miR-145-5p, LC3B, Beclin-1	[120]
	Mice with left ureteral ligation Ang II-induced HK-2 cells	6.25, 12.5, 25 mg/kg 0.1, 1, 10 µM	Intraperitoneal injection	Decrease: Serum creatinine, Blood urea nitrogen, TGF-β1, FGF-2, SDC1, α-SMA Increase: HPSE, E-cadherin	[125]
	Rat with the left ureter was exposed via a left flank incision and ligated Human serum albumin induced the HK-2 human renal proximal tubular cell line	12.5 mg/kg 20 μM/L	Oral administration	Decrease: β2-microglobulin, N-Acetamidoglucosidase, Blood urea nitrogen, Serum creatinine, CTGF, PDGF-C, PDGFR-α, caspase-3, Early growth response protein 1, CHOP, GRP78 Increase: Par-3	[126]
	High-fat diet fed in mice Palmitic acid-, tunicamycin-, and thapsigargin-induced human proximal tubule epithelial HK-2 cells	3, 6.25, 12.5 mg/kg 1, 10, 100 μM	Intraperitoneal injection	Decrease: ICAM-1, VCAM-1, IL-1β, IL-6, TNF-α, Bax, cleaved caspase-3, BIP, P-eIF2α, ATF4, CHOP, ATF6, IRE1α, XBP1s Increase: Bcl-2	[127]
	db/db mice fed a high-calorie diet	50, 100, 150 mg/kg	Gavage administration	Decrease: p-Smad3/Smad3, Collagen type IV, TGF-β1, IL-6, TNF-α, Urine microalbumin, Urine creatinine, Blood urea nitrogen, TC, TG	[128]
	High-glucose and high-fat feeding combined with streptozotocin-induced rats High-glucose-induced glomerular-tethered HGMC cells	80, 160 mg/kg 10^−7^, 10^−6^, 10^−5^ Mol/L	Gavage administration	Decrease: Fasting blood glucose, TG, TC, Urea nitrogen, Urine microalbumin, Serum creatinine, MDA, NO, Collagen content, TGF-β1, Phosphorylated Smad2, Phosphorylation of p38MAPK, Collagen I, Collagen III, Fibronectin, Laminin, α-SMA, Smad2, p38MAPK Increase: Smad7, Albumin, Total protein	[129,130]

**Table 7:** Effects of salvianolic acid B on the intestine.

Pharmacology	Experimental Subject	Experimental Dose	Animal Experimental Drug Delivery Method	Effects	Ref.
Effects on the intestine	H_2_O_2_-induced rat small intestine IEC-6 cells Ischemia/reperfusion injury of rat small intestinal mucosa	10, 50, 100, 500 μM 40 mg/kg	Intraperitoneal injection	Decrease: Apoptosis, Mitochondrial membrane potential depolarization, ROS, Cyt-c, caspase-3, Bax Increase: Bcl-2	[132,133]
	Clamping of the superior mesenteric artery in rats	40 mg/kg	Intraperitoneal injection	Decrease: MDA, IL-1β, IL-6, TNF-α, MPO, NF-κB p65 Increase: PI3Kp85α, p-AKT (ser473), SOD, CAT, GSH	[134]
	Intestinal ischemia/reperfusion injury in rats	40 mg/kg	Intraperitoneal injection	Decrease: MDA, TNF-α, IL-1β, IL-6, NF-κB Increase: SOD	[135]
	Mice with free drinking water containing dextran sulfate sodium and injected with IL-1β	80, 100 mg/kg	Gavage administration	Decrease: MLCK	[136]

**Table 8:** Effects of salvianolic acid B on bone.

Pharmacology	Experimental Subject	Experimental Dose	Animal Experimental Drug Delivery Method	Effects	Ref.
Effects on the bone	H_2_O_2_-induced extraction of nucleus pulposus cells from rat caudal intervertebral discs Induction of degeneration of the caudal intervertebral disc in rats by percutaneous needling	0.001, 0.01, 0.1, 1, 10, 100 nM 200 mg/kg	Gavage administration	Decrease: ROS, MDA, Bax, Cleaved caspase 3, IL-1β, IL-6, TNF-α, MMP3, MMP13, ADAMTS4, ADAMTS5 Increase: SOD, CAT, GSH-Px, Bcl-2, Collagen II, Aggrecan	[137,138]
	Aspiration of the nucleus pulposus in New Zealand white rabbits	0.01, 0.1, 1, 10 mg/L	Injection passed through the annulus fibrosus	Increase: Runx2, Sox9, Collagen II, Proteoglycan content, TGF-β1	[139]
	Expand the premaxillary suture of rats	40 mg/kg	Intraperitoneal injection	Increase: Vascular, Osteoblasts, Osteoclasts	[140]
	Human bone marrow mesenchymal stem cells Bilateral ovarian removal combined with sawing of the femur in rats	0.5, 1, 5 μM 2.5 mg/kg	Local injection	Increase: Endomucin, Hypoxia-inducible factor 1-α, OPN	[141]
	Extraction of mesenchymal stem cells from human bone marrow	5 μM	None	Decrease: ROS	[142]
	Mouse osteoblasts MC3T3-E1	0.01, 0.03, 0.10, 0.30, 1.00 nmol/L	None	Increase: OPN, OCN, Collagen I, ALP, PI3K, p-AKT	[143]
	Prednisolone acetate-induced osteoblasts isolated from the skull of rats	None	None	Decrease: Nrf2 Increase: Runx2, Osx, OCN, IGF-I, Collagen I, HO-I	[144]
	Human gingival mesenchymal stem cells	0, 5, 10 μMol/L	None	Decrease: PPARγ, C/EBPα Increase: Runx2, OCN, Collagen I, ALP	[145]
	H_2_O_2_-induced periodontal ligament stem cells in orthodontic patients	0.5, 1, 5, 10 μMol/L	None	Decrease: PPARγ, LPL, C/EBPα, ROS Increase: ALP, Collagen 1, RUNX2, OCN, Akt, SOD, GSH, Nrf2	[136]
	Human periodontal ligament cell tissues were isolated from periodontal ligament of the middle third of healthy molars	0.1, 0.5, 1, 5 μM	None	Increase: RUNX2, BMP2, OSX, OCN, β-catenin, LEF1, Cyclin D1, ALP, Phosphorylated GSK-3β	[147]
	High-fat diet feeding and anterior cruciate ligament transection/medial meniscus removal in mice Palmitic-acid-induced mouse chondrocyte cell line ATDC5	25 mg/kg 25, 50, 100 μM	Intraperitoneal injection	Decrease: TNF-α, IL-6, PGE2, JAK2, STAT3 Increase: KCNQ1OT1, Bcl-2, LC3II/LC3I, Beclin-1	[148]

**Table 9:** Effects of salvianolic acid B on the liver.

Pharmacology	Experimental Subject	Experimental Dose	Animal Experimental Drug Delivery Method	Effects	Ref.
Effects on the liver	Alcoholic liquid feed for rats	30, 15, 30 mg/kg	Gavage administration	Decrease: ALT, AST, TG, TC Increase: Ethanol dehydrogenase, SIRT1, p-AMPKα1/AMPKα1	[149]
	Ethanol-induced HepG2 cells	8 μMol/L	None	Increase: Cell survival rate, p-AMPKα1/AMPKα1	[150]
	High-fat-diet-induced ApoE knockout mice	15 mg/kg	Intraperitoneal injection	Decrease: AST, ALT, TNF-α, NF-κB, IL-6, Nrf2, HO- 1 Increase: Beclin1, p62, LC3, AMPK	[151]
	Arsenic-trioxide-induced HepaRG human hepatocytes	10, 5, 2.5 μMol/L	None	Decrease: Apoptosis Increase: Cell survival, Mitochondrial membrane potential, Bcl-2/Bax, p-Akt/Akt	[152]
	Arsenic-trioxide-induced rat	5, 10, 20, 40 mg/kg	Intraperitoneal injection	Decrease: MDA Increase: SOD	[153]
	Mice injected with CCl_4_ H_2_O_2_-induced rat hepatic stellate cells HSC-T6	7.5, 15, 30 mg/kg 25, 50, 100 μMol/L	Gavage administration	Decrease: ALT, AST, MDA, pSmad2C, pSmad2L Increase: SOD, GSH	[154,155]
	Senecionine-induced mice	10 mg/kg	Gavage administration	Decrease: GSH, PAI-1, MMP-9, TGF-β1 Increase: SOD	[156]
	α-Naphthyl isothiocyanate-induced rat	15, 30 mg/kg	Intraperitoneal injection	Decrease: ALT, ALP, AST, γ-glutamyl transferase, Total bilirubin, Direct bilirubin, Total bile acids, IL-1β, IL-6, TNF-α, TGF-β, COX-2 Increase: Cyp3a2, Ugt1a1	[157,158]
	A cecal ligation and puncture method to induce mice sepsis	30 mg/kg	Intraperitoneal injection	Decrease: TNF-α, IL-6, Bax, AST, ALT, MPO, caspase-3 Increase: Bcl-2, SIRT1, PGC-1α	[159]
	Cyclophosphamide-induced Nile tilapia	0.25, 0.50, 0.75 g/kg	Mixed with feed for oral administration	Decrease: ROS, Atg-3, -5, -7, -13, Beclin1, MP-activated protein kinase, unc-51-like autophagy-activating kinase 1, mucolipin-1, lysosomal associated membrane protein 1, UV-radiation-resistance-associated gene, transcription factor EB, caspase-3, -8, and -9, Cytc, Bax, p53, JNK1, 2, apoptosis signal-regulating kinase 1, p38, extracellular signal-regulated protein kinase Increase: SOD, GSH, CAT, NO, Gpx3, glutamate–cysteine ligase catalytic subunit, glutathione reductase, glutathione s-transfer α, catalase, nrf2, uncoupling protein 2, heme oxygenase-1, p62, mTOR, Bcl-2	[160]
	Diethylnitrosamine-induced mouse TGF-β1-induced HSC-T6 cells	25, 50, 100 μMol/L 15, 30 mg/kg	Gavage administration	Increase: Cleaved caspase-9, Apoptosis	[161,162]
	Diethylnitrosamine-induced mouse TGF-β1-induced HSC-T6 cells and LX-2 cells	25, 50, 100 μMol/L 15, 30 mg/kg	Gavage administration	Decrease: α-SM, TGF-β1, Collagen I, P-Smad2C, P-Smad2L, P-ERK1/2, P-JNK1/2, P-p38, P-Smad3C, P-Smad3L, Plasminogen activator inhibitor 1	[163]
	Carbon-tetrachloride-induced rats	60 mg/kg	Oral administration	Decrease: TGF-β1, Shh, Ptch1, Smo, Gli1, α-SMA, ALT, AST, Total bilirubin Increase: Albumin	[164]
	LPS-induced human hepatic stellate cell line LX-2	1.0–5.0 μM	None	Decrease: Cell activation, Cell proliferation Increase: FGF19, FGFR4	[165]
	TGF-β1-induced mouse immortalized stellate cell lines JS1 TGF-β1-induced human hepatic stellate cell lines LX2	10^−5^ M	None	Decrease: LC3B II, Collagen Ⅰ, α-SMA, Atg5 Increase: C-Caspase 3	[166]
	Rats injected with dimethylnitrosamine TGF-β1-induced hepatic stellate cells from liver tissue and rats	12.5 mg/kg 1 μM	Gavage administration	Decrease: MEF2, α-SMA, Collagen I	[167]

**Table 10:** Effects of salvianolic acid B on the skin.

Pharmacology	Experimental Subject	Experimental Dose	Animal Experimental Drug Delivery Method	Effects	Ref.
Effects on the skin	Human immortalized cells HaCaT	3.125, 6.25, 12.5 μMol/L	None	Decrease: Cell proliferation, Bcl-2 Increase: Apoptosis, Caspase3, Caspase9, Bax	[168]
	Bleomycin-induced mice TGF-β-induced human skin fibroblasts	10 mg/kg 12, 25, 50 μg/mL	Intraperitoneal injection	Decrease: Collagen content, Connective tissue growth factor, Profibrotic gene: FN1, Plasminogen activator inhibitor-1, α-SMA, phosphorylation of SMAD3, phosphorylation of ERK1/2	[169]
	Imiquimod-induced mice psoriasis	40 mg/kg	Intraperitoneal injection	Decrease: Values of psoriasis area, IL-22, IL-23, IL-17A, IL-1β, IL-6, MDA, Keratin markers (K16 and K17), pAkt/Akt, pPI3K/PI3K Increase: SOD, CAT	[170]

**Table 12:** Combination therapy of salvianolic acid B.

Combination Therapy with	Test Subject	Mechanism/Effect	Ref.
Ginsenoside Rg1	Rat with ligature below the left descending coronary artery	Decrease: Infarct area, Inflammatory infiltration, Collagen volume fraction, Fibrosis, Collagen I/III, α-SMA, MMP-9	[192]
	High-fat-diet-induced mice	Decrease: TC, TG, LDL-c, Non-esterified fatty acid	[193]
Sodium tanshinone IIA sulfonate	Foponate combined with LPS-induced THP-1 macrophages TGF-β1-induced MRC-5 cells	Decrease: IL-1β, TNF-α, α-SMA, Collagen 1α1, Fibronectin	[194]
Tanshinone IIA	Bovine serum albumin-induced myocardial necrosis in mice	Decrease: Creatine kinase, CK-MB, LDH, α-Hydroxybutyrate dehydrogenase, TNF-α, IL-6, IL-1β, AST	[195]
Hydroxysafflor yellow A	Oxygen–glucose deprivation/reperfusion-induced H9c2 cardiomyocyte	Decrease: TNF-α, IL-6, IL-1, MDA, LDH, Creatine kinase Increase: SOD	[196]
Magnesium salt Salvia Miltiorrhiza Polyphenolate Injection	HEK293T cells, human hepatocellular carcinoma Huh-7 cells, 293T cells stably overexpressing human-derived SAR-CoV-2 receptor protein ACE2	Anti-SARS-CoV-2	[197]

**Table 13:** New dosage forms and novel routes of administration of salvianolic acid B.

Dosage Form	Preparation	Test Subject	Drug Delivery Routes	Effects	Ref.
Nanosheet	Graphitic carbon nitride nanosheets were prepared using an alkali–chemical–ultrasonic-assisted exfoliation method, added with PEI aqueous solution to obtain PEI–graphitic carbon nitride nanosheets	Mouse fibroblast L929 Wild AB lineage zebrafish	None	Increase: Slow release performance, Drug delivery rate	[198]
Nanoparticles	Chitosan powder was added to deionized water, glacial acetic acid was added and dissolved by sonication, an aqueous solution of carboxymethyl panax notoginseng polysaccharides was added, and the precipitate was collected and lyophilized	Mouse breast cancer cells 4T1 Mouse cardiomyocytes H9C2	None	Sustained release	[199]
Nanoparticles	Salvianolic acid B and poloxamer 188 were dissolved by sonication in distilled water; ice chips and 1.2% n-butyl cyanoacrylate were dissolved with an appropriate amount of acetone and added dropwise to the above solution	Mice	Intravenous injection	Increase: Brain targeting	[200]
Nanoparticles	Dexamethasone−salvianolic acid B conjugate 1 was dissolved in ethanol and added to phosphate-buffered saline (PBS). Subsequently, the ethanol was removed under vacuum to give conjugate 1 nanoparticles	Auditory hair cell line HEI-OC1 Zebrafish Cisplatin-induced guinea pig	Transtympanically administered	Decrease: Ototoxicity	[201]
Nanoparticles	Free salvianolic acid B embedding phospholipid complex nanoparticles, free salvianolic acid B, and salvianolic-acid-B–phospholipid complex-loaded nanoparticles	4-nitroquinoline-N-oxide-induced mice	Orally administered	Decrease: Cell proliferation (Ki67 and PCNA) markers, Cell cycle (cyclin D1 and p16) markers	[202]
Composite porous scaffold	PLGA and β-TCP were prepared by dissolving in 1,4-dioxane, and salvianolic acid B was dissolved in 1,4-dioxane. Then, different volumes of salvianolic acid B solution were added and entirely dried in a freeze-dryer	EA-hy9.26 endothelial cells Rat subperiosteal dissection with elevation of all paraspinal muscles and periosteum from the posterior elements was performed to expose the L5 and L6 transverse processes of the rat, and implanted with the same size of PLGA/β-TCP	Intracorporeal implantation	Increase: Bony fusion, Osteogenesis, Migration, Angiogenesis	[203]
Composite scaffold	GO was ultrasonically dispersed in dioxane/water, the solution was added with PLA, and different amounts of salvianolic acid B were then added. The homogeneous solution was poured into a mold until gel formed and finally frozen	Osteoblast cells MC3T3-E1	None	Increase: Bone regeneration, Blood vessel formation	[204]
Scaffold	GO/salvianolic acid B conjugates and GO were dissolved in ddH_2_O with silk fibroin, added to a mold, and placed in a freeze-dryer	EA-hy9.26 endothelial cell Rat BMSCs Rat cranial defect model implantation of scaffolds	Intracorporeal implantation	Increase: Cell migration, Osteogenesis, Angiogenesis, Bone regeneration	[205]
Scaffold	Chitosan was added to 2% acetic acid, and salvianolic acid B was dissolved in ethanol and added to the chitosan solution. Then, Ca (NO3)2 and KH_2_PO_4_ were added to prepare the salvianolic acid B–chitosan/hydroxyapatite precursor solution. The solution was poured into a plastic mold and freeze-dried	Rabbit radius defect MC3T3-E1 cells HUVECs	Intracorporeal implantation	Increase: Vessel area, Vessel size	[206]
Biomimetic composite scaffolds	GO and ASA were ultrasonically dispersed in a dioxane/water mixture, and PLA was successively added to the mixed solution. Then, salvianolic acid B was added. The solution was poured into the mold until the solution formed a gel and was then frozen	Pre-osteoblast cells MC3T3-E1	None	Decrease: Platelet aggregation Increase: Cell proliferation	[207]
Hydrogel	Peptide was dissolved in PBS, and transglutaminase stock solution with calcium chloride and dithiothreitol was added to form a hydrogel. A polydopamine nanoparticle solution was added to salvianolic acid B solution	HUVEC Rat coronary artery ligation	Intramyocardial injection	Decrease: Ventricular remodeling	[208]
Hydrogel	1% HAMA hydrogel as a carrier and co-encapsulated BMSCs and salvianolic acid B into the hydrogel	BMSCs Needling segments 7–10 of the rat caudal spine using a 21G puncture needle	Inject into the intervertebral disc	Decrease: Apoptosis, Nucleus pulposus loss, Annulus fibrosus rupture disorder	[209]
Hydrogel	Photoinitiator LAP was added to PBS and dissolved. Salvianolic acid B was dissolved in PBS and added to the methacrylate-based gelatin hydrogel precursor solution to form a gel	Needling segments 7–10 of the rat caudal spine using a 20G puncture needle	Inject into the intervertebral disc	Decrease: TLR4, NF-kB, MMP3, MMP13, ADAMTS4, ADAMTS5, Collagen II, Proteoglycan	[210]
Hydrogel	Phospholipids, cholesterol, and tanshinone IIA were dissolved in dichloromethane. Salvianolic acid B was dissolved in 1% glycine–hydrochloric acid buffer solution and added to the above solution. The lyophilized powder of tanshinone IIA-salvianolic acid B was redissolved in oxidized hyaluronic acid oxidized hyaluronic acid solution, added to N-succinyl chitosan, and stirred	Rat abdominal skin New Zealand rabbit	Ex vivo dermal administration	Increase: Transdermal absorption, Drug dermal retention	[211,212]
Liposomes	Glycol chemically coupled to the membrane penetrating peptide TAT dissolved in an organic solvent, salvianolic acid B was dissolved in a 1% glycine–hydrochloric acid buffer solution. The two-phase system was mixed to form a water-in-oil emulsion, and then extruded with liposome extruder	Human skin fibroblast (HSF) cells Rat	Ex vivo dermal administration	Decrease: Cell proliferation, Cell migration, Cell invasion	[213,214,215]
Liposomes	Soy lecithin, cholesterol, and tanshinone IIA were dissolved in chloroform. Salvianolic acid B was dissolved in 1% glycine–HCl buffer solution and added dropwise to tanshinone IIA liposomes, obtain the liposome suspension, and extruded with liposome extruder	Human skin fibroblast HSF cells Rat	Ex vivo dermal administration	Decrease: Cell proliferation, Cell migration, Cell invasion, Collagen I Increase: MMP2	[216]
Liposomes	Cholesterol, distearoyl phosphatidylethanolamine–polyethylene glycol 2000, and egg yolk lecithin were dissolved in anhydrous ethanol, and salvianolic acid B was dissolved in ultrapure water. The anhydrous ethanol mixture was injected into the drug-containing ultrapure water	Mouse mammary carcinoma 4T1 cells and mouse embryonic fibroblast NIH3T3 cells injected into mice	Intravenous injection	Decrease: α-SMA, TGF-β1, Smad2, Smad3, pSmad2, pSmad3, Smad4 Increase: IFN-γ, TNF-α, IL-12α, IL-2, IL-4, IL-6, IL-10	[217]
Lipid emulsion	The medium-chain triglycerides and natural lecithin were heated and sonicated in a water bath; the aqueous phase was added to the oil phase and sheared at high speed for 2 min, and placed in an ultrasonic cell disruptor with fixed power	Acetaminophen-induced mouse	Gavage administration	Decrease: MDA, ALT, AST	[218]
Dry powder	Upon dissolving salvianolic acid B and L-leucine in water, the spray would begin	Mouse embryonic fibroblast NIH-3T3 Bleomycin saline solution-induced rat	Noninvasive tracheal administration	Decrease: Collagen 1A1, Collagen 3A1, ICAM-1, FN, iNOS, Arg-1, Cell migration, MDA, MPO, TGF-β1, IL-1β, IL-4, IL-6, IL-18 Increase: IFN-γ, SOD	[219]
Microemulsion	The oil phase was mixed with a surfactant–cosurfactant mixture. And the mixture was titrated with PBS buffer until it turned turbid	Mice skin Imiquimod-induced mice	Applied to the dorsal skin	Decrease: IL-17A, IL-17F, IL-22, IL-23, PCNA	[220]
